# Desmoglein-2 Affects Vascular Function in Moyamoya Disease by Interacting with MMP-9 and Influencing PI3K Signaling

**DOI:** 10.1007/s12035-024-04010-0

**Published:** 2024-02-07

**Authors:** Ajun Wang, Nan Li, Nan Zhang, Jian Liu, Tao Yang, Dongxue Li, Changwen Li, Rui Li, Tongcui Jiang, Chengyu Xia

**Affiliations:** 1 Department of Neurosurgery, The First Affiliated Hospital of USTC, Division of Life Science and Medicine, University of Science and Technology of China, 17 Lujiang Road, Hefei, Anhui Province, China; 2 Department of Neurosurgery, Anhui Provincial Hospital, Affiliated to Anhui Medical University, Hefei, China; 3 School of Basic Medical Sciences, Anhui Medical University, 81 Meishan Road, Hefei, Anhui Province China

**Keywords:** Apoptosis, DSG2, Hypoxia, Moyamoya disease, Vascular remodeling

## Abstract

The pathogenesis and development of Moyamoya disease are still unclear. This study aimed to investigate the effect of desmoglein-2 (DSG2) on Moyamoya disease and determine the inhibitory effect of DSG2 in vascular remodeling in Moyamoya disease.

RNA sequencing, immunohistochemistry (IHC), and western blotting were used to detect the expression of DSG2 in the superficial temporal artery (STA) tissues of Moyamoya disease. The association between DSG2 and endothelial cells’ biological activities was investigated by cell counting kit-8 (CCK-8), migration assay, tube formation assay, flow cytometry with Annexin V-FITC/PI staining, and TUNEL apoptotic cell detection kit. Pathways affected by overexpression or knockdown of DSG2 were identified in endothelial cells.

The expression of DSG2 in the STA tissues of Moyamoya disease was lower than that in normal controls. Overexpression of DSG2 inhibits the proliferation and migration but promotes apoptosis in endothelial cells, and low DSG2 levels result in impaired angiogenesis. In addition, there was an interaction between DSG2 and MMP-9, and DSG2 acted through the PI3K signaling in endothelial cells.

Our results indicate that DSG2 affects PI3K signaling in vascular endothelial cells, and MMP-9 is involved in DSG2-mediated vascular changes in Moyamoya disease.

**Supplementary Information:**

The online version contains supplementary material available at 10.1007/s12035-024-04010-0.

## Introduction

Moyamoya disease is a chronic occlusive cerebrovascular disease, which is characterized by progressive stenosis or occlusion of the terminal internal carotid artery (ICA) and its proximal branches, accompanied by the formation of abnormal collateral vessels [1, 2]. The incidence of Moyamoya disease has obvious geographical differences, which is more common in Japan, China, Korea, and southeastern Asian countries [3]. The main clinical manifestation of Moyamoya disease is unpredictable hemorrhagic or ischemic stroke, which causes high mortality and disability rates [4]. The pathogenesis of Moyamoya disease has not been elucidated, which may involve angiogenesis, genetic factors, and immune and inflammatory responses [4, 5]. Recently, some reports [6, 7] have described impaired cytoskeletal regulation, and fibroplasia of smooth muscle cells may be the potential etiology.

Desmogleins (DSG) are a family of cadherin adhesion proteins which were first found in desmosomes. They provide cardiomyocytes and epithelial cells with the junctional stability to tolerate mechanical stress [8]. One of its members, DSG2, is expressed by non-desmosome-forming human endothelial progenitor cells as well as their mature counterparts endothelial cells (ECs) [9]. DSG2 not only plays an important role in the migration and invasion of cancer cells and the formation of tumor blood vessels but also actively coordinates signaling pathways, thereby mediating proliferation, differentiation, and apoptosis [10–12]. In addition, DSG2 is also expressed by cells of endothelial cell lines and contributes to the formation of new blood vessels [9].

However, the role of DSG2 in Moyamoya disease has never been explored. In this study, we used RNA sequencing, Western blot, and immunohistochemistry to analyze DSG2 levels in the vessels of Moyamoya disease.

In addition, we concluded that DSG2 is protective for vascular endothelial cells via PI3K signaling, and MMP-9 is involved in DSG2-mediated vascular changes in Moyamoya disease.

## Methods

### Patients and Tissue Specimens

As a systemic vascular disease, the pathological manifestations of extracranial and intracranial vessels of Moyamoya disease are essentially the same [13], and it is not easy to obtain the intracranial lesion vessels of Moyamoya disease, so we used the superficial temporal artery (STA) tissues trimmed during bypass surgery for related research. The STA tissues were collected from patients with Moyamoya disease who underwent combined direct and indirect bypass surgery and patients with brain trauma requiring craniotomy in the Department of Neurosurgery, First Affiliated Hospital of USTC (Anhui Provincial Hospital). One part was fixed in a 10% neutral formalin solution, and the other part was stored in a refrigerator at − 80 ℃. All protocols using human specimens were approved by the ethics committee of the First Affiliated Hospital of USTC (Anhui Provincial Hospital). Written informed consent was obtained from all patients. All protocols were approved by the Institutional Review Board of the First Affiliated Hospital of USTC (Anhui Provincial Hospital).

### RNA Sequencing

Total RNA was extracted from the STA tissues using Trizol reagent (Invitrogen, Carlsbad, CA, USA) according to the manufacturer’s protocol. The integrity and concentration of RNA were detected using an Agilent Bioanalyzer 2100 system (Agilent Technologies, Santa Clara, CA, USA), enriched and purified with Oligo (dT)-bearing magnetic beads. RNA sequencing was performed by Anoroad (Beijing, China). All sequencing reads were exported in FASTQ format. RNA sequencing results have been submitted to the Gene Expression Omnibus database (GSE249254).

### Immunohistochemistry

STA tissue slides (3 µm) were immersed in xylene at 50 ℃ for 40 min, followed by 100% ethanol for 3 min, 95% ethanol for 3 min, 90% ethanol for 3 min, 85% ethanol for 3 min, 80% ethanol for 3 min, 75% ethanol for 3 min, and ddH_2_O for 3 min; immersing the slices in 0.01 M citric acid buffer solution, heating for 5 min with the maximum firepower (98–100 °C) in a microwave, cooling (about 3 min), and repeating once; washing with PBS. Peroxidase inhibitor was added dropwise at room temperature for 10 min and washed with PBS. Rabbit anti-human DSG2 (Abcam, #ab150372) monoclonal antibody (1:200) was added to each specimen section, overnight at 4 ℃, rewarming at 37 ℃ the following day, washing with PBS, adding enzyme-labeled goat anti-mouse/rabbit IgG polymer dropwise, 20 min at 37 ℃; washing with PBS; the newly prepared DAB developer was added to the slices, and the reaction time (about 5 min) was detected under the microscope at room temperature; deionized water was used; hematoxylin was used for counterstaining, and tap water was used for washing; the slices were directly dried in an oven, and transparent xylene was used overnight; the slices were sealed with gum, and pictures were taken with a microscope. ImageJ software was used to analyze the area ratio of the positive area.

### Cell Culture

The hCMEC/D3 cells were provided by the Institute of Intelligent Pathology, First Affiliated Hospital of USTC (Anhui Provincial Hospital). Human umbilical vein endothelial cells (HUVECs) and human embryonic kidney cells (293 T) were provided by the Department of Biochemistry and Molecular Biology, Anhui Medical University. All cells were validated by the STR genotype test. The hCMEC/D3 cells and HUVECs were cultured in ECM (ScienCell, USA), The 293 T cells were cultured in DMEM medium (Cytiva, USA), and all cells were used for experiments. All cells were maintained in 5% CO_2_ and 95% relative humidity at 37 °C.

### Plasmid Transfection

The plasmids of NC-pEGFP-C1 and DSG2-pEGFP-C1 were constructed by Sangon Biotech (Shanghai, China). The plasmid was transfected into the cells by Lipofectamine 2000 (Invitrogen, USA) according to the instructions of the reagent supplier. After 24–48 h of cell culture, the plasmid was transfected into the cells and successfully expressed by Western blot and qPCR verification.

### siRNA‑Mediated Knockdown of DSG2

HUVECs were transfected with control siRNA (GenePharma, China) or DSG2-specific siRNA (GenePharma, China) using GP-transfect-Mate (GenePharma, China) and Opti-MEM media at the time of cell culture. The DSG2 siRNA sequences were 5′-GCCCAUGCAAGAUAUGUAATT-3′ (forward) and 5′-UUACAUAUCUUGCAUGGGCTT-3′ (reverse).

### Hypoxia Induction

Cobalt chloride (CoCL_2_) stimulation of HUVECs is often used as an in vitro hypoxia model for studies. Hypoxia induction was performed using CoCL_2_ (Aladdin, Shanghai, China) hexahydrate. After HUVECs were grown to 70–80% density, different concentrations (0 µM, 10 µM, 50 µM, 100 µM, 200 µM, 300 µM) of CoCL_2_ were added, cultured in 5% CO_2_ and 95% relative humidity at 37 °C, and protected from light. Cellular proteins were extracted 24 h later for Western blot. The concentration of CoCL_2_ that inhibited the DSG2 level most significantly was selected for the time gradient, and the cells were collected at different time points (0 h, 3 h, 6 h, 12 h, and 24 h) to extract cell proteins for Western blot, and finally, the cobalt chloride concentration that inhibited the DSG2 level most significantly and the hypoxia induction time were selected for subsequent related experiments.

### Western Blot

The samples were subjected to SDS-Page with a PAGE gel (Yazyme, Shanghai, China). Five percent skim milk was blocked for 1 h at room temperature and washed with PBST 3 times for 10 min each. Incubated with primary antibody (4 ℃, overnight), washed with PBST three times for 10 min each, and secondary antibody (room temperature, 1.5 h), washed with PBST 3 times for 10 min each, and the spots were visualized by enhanced chemiluminescence using a protein imaging system (Qin Xiang, Shanghai, China). For the quantification of changes in protein levels, densitometric analysis was performed via ImageJ software. Antibodies are detailed in the Supplemental Digital Content.

### RNA Extraction and Quantitative Real‑Time Polymerase Chain Reaction (qPCR)

RNA was extracted from cells and STA tissues using *RNAex Pro* Reagent (#AG21102, Accurate Biology, Hunan, China) according to the manufacturer’s instructions. RNA samples were reversely transcribed using *Evo M-MLV* RT Premix for qPCR (#AG11706, Accurate Biology, Hunan, China) kits following the manufacturer’s instructions. QPCR was used to quantify the gene expression level. QPCR amplification was performed on a real-time fluorescence quantitative PCR instrument using SYBR® Green Premix *Pro Taq* HS qPCR kit (#AG11701, Accurate Biology, Hunan, China). Reaction parameters: react at 95 ℃ for 30 s, then cycle for 5 s at 95 ℃ and 30 s at 65 ℃ for 45 cycles, then form a molten phase. Glyceraldehyde-3-phosphate dehydrogenase (GAPDH) was used as an internal control to standardize the Cq value of each group, and then the relative expression of different genes in different samples was calculated by the 2^−△△Ct^ method. The results shown for all mRNA levels were obtained by quantizing three or more independent biological replicas. Primers are detailed in the Supplemental Digital Content.

### Cell Proliferation Assay

Cell proliferation was detected by a CCK-8 cell counting kit (biosharp, Hefei, China). Cells were seeded into 96-well plates with an initial density of 2000 cells per well. Overnight, 10 µL of CCK-8 was added to each well after the cells were adherent. Incubation was continued, and the absorbance (450 nm) was measured with a spectrophotometer after 3, 6, 12, 24, 48, and 72 h.

### Cell Migration Assay

Transwell migration assay and wound-healing assay were used to detect the migration ability of cells. Transwell chambers (Corning Costar, Cambridge, MA) were placed in a 24-well plate, and ECM medium containing 5% fetal bovine serum (FBS) was then added as a chemical inducer to the 24-well plate over the bottom of the Transwell chamber. Cells were digested with trypsin and resuspended in an FBS-free ECM medium. Cell suspension (200 µl) at a concentration of 1.5 × 10 [4] cells/well was loaded into the upper well, incubated at 37 ℃ for 48 h, and the cells were fixed and stained with crystal violet. Cells migrating to the opposite side of the filter were photographed with a light microscope, and cell numbers were counted manually using ImageJ software. The cells were cultured in 6-well plates. When the cells grew to 90% density, they were scratched manually with a 100-µl sterile pipette, washed twice with PBS, and then replaced with serum-free ECM medium for 24 and 48 h. Wound margins in the same area were photographed under a microscope. The gap area was measured using ImageJ software.

### Cell Tube Formation Assay

Matrigel (ABW, Shanghai, China) was dissolved overnight at 4 ℃, 50 µL/well was coated in a pre-coated 96-well plate to avoid bubbles in the addition process. It was placed in an incubator at 37 ℃ for 30 min and solidified for later use. Cells were digested with trypsin and resuspended in DMEM medium containing 10% FBS, and 50 µL of resuspension was added to each well at a concentration of 4 × 10 [4] cells/well, repeated in three wells, incubated in 37 ℃ incubator, and then photographed under the microscope after the cells formed tubes.

### Apoptosis Assay

The apoptosis level was detected by Annexin V-FITC/PI double labeling apoptosis detection kit (BestBio, Shanghai, China) and TUNEL apoptosis detection kit (Beyotime Institute of Biotechnology, Jiangsu, China) according to the protocol of the reagent manufacturer. The apoptosis level was determined by analyzing the percentage of annexin V-positive cells and the percentage of TUNEL-positive cells. STA endothelial cells were assayed for the level of apoptosis using a chromogenic TUNEL apoptotic cell detection kit. The apoptotic index was calculated as the percentage of TUNEL-positive cells relative to total cells.

### Co-immunoprecipitation

Co-immunoprecipitation (Co-IP) was used to detect proteins that interact with DSG2. After successful cell transfection, immunoprecipitation of DSG2 protein was performed according to the manufacturer’s instructions (Pierce™ Classic Magnetic Bead Immunoprecipitation/Co-Immunoprecipitation Kit, Thermo Scientific, USA), and after elution of the antigen–antibody complex, the samples were subjected to Western blot analysis.

### Immunofluorescence

After cells were cultured on slides to attachment, they were fixed with 4% paraformaldehyde for 20 min, washed three times with PBS, then permeabilized with 0.5% TritonX-100 for 20 min, and washed three times with PBS. After blocking with 5% BSA for 1 h, they were incubated with primary antibodies overnight at 4 °C and then washed three times with PBS followed by incubation with secondary antibodies for another 1 h. Slides were removed and inverted onto slides containing an anti-fluorescence quenching mounting medium, and images were taken under an upright fluorescence microscope.

### Statistical Analysis

GraphPad Prism 9.4 statistical software was used for data analysis. All results are expressed as mean ± SEM. T-test was used for comparison between two groups, and one-way ANOVA was used for comparison between two and more groups. *P* < 0.05 is considered statistically significant.

## Results

### DSG2 Levels Decreased in Moyamoya Disease–Related STA Endothelial Cells

**Fig. 1:**
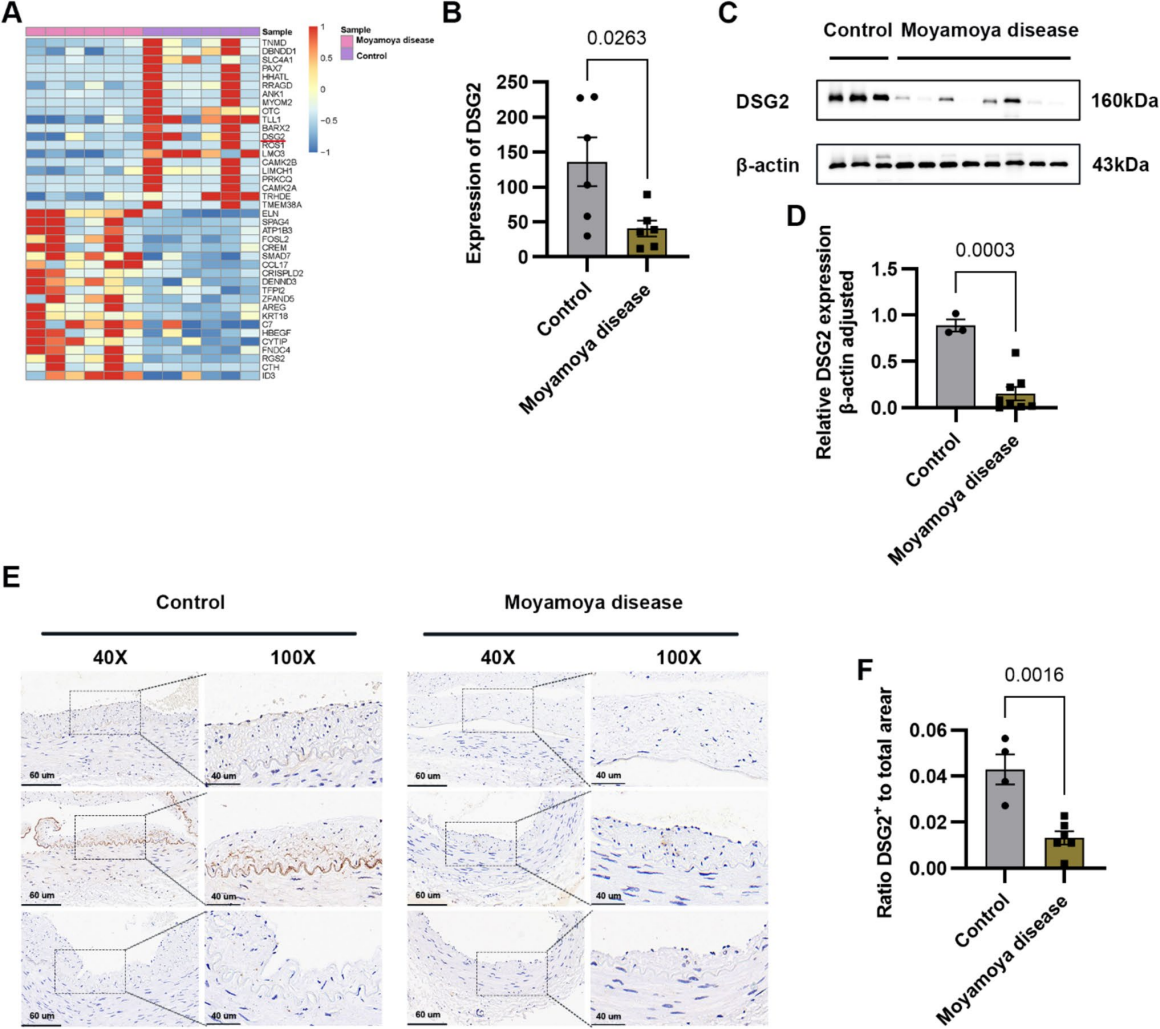
Downregulation of DSG2 expression in Moyamoya disease.** A**, **B** RNA sequencing of the STA in Moyamoya disease and normal controls showed that DSG2 expression decreased in Moyamoya disease; **C**, **D** Western blot analysis of DSG2 protein levels in Moyamoya disease and normal control STA; **E**, **F** immunohistochemical analysis showed that DSG2 was expressed in the endothelial cells and intima of the STA, and decreased in Moyamoya disease. Scale bar: 60 µm and 40 µm. DSG2, desmoglein-2

RNA sequencing showed that the mRNA level of DSG2 was significantly decreased in Moyamoya disease compared with normal controls (Fig. 1A, B). To verify this sequencing result, we first collected tissues from Moyamoya disease and performed a Western blot. The results of Western blot showed that the expression of DSG2 protein in Moyamoya disease was significantly reduced (Fig. 1C, D). The results of IHC showed that the STA tissue intima of Moyamoya disease was significantly thicker than that of normal control, and the expression of DSG2 was also significantly decreased (Fig. 1E, F). These results suggest that DSG2 expression was downregulated in the Moyamoya disease condition.

### DSG2 Expression Inhibits Cell Proliferation and Migration But Promotes Tube Formation In Vitro

**Fig. 2:**
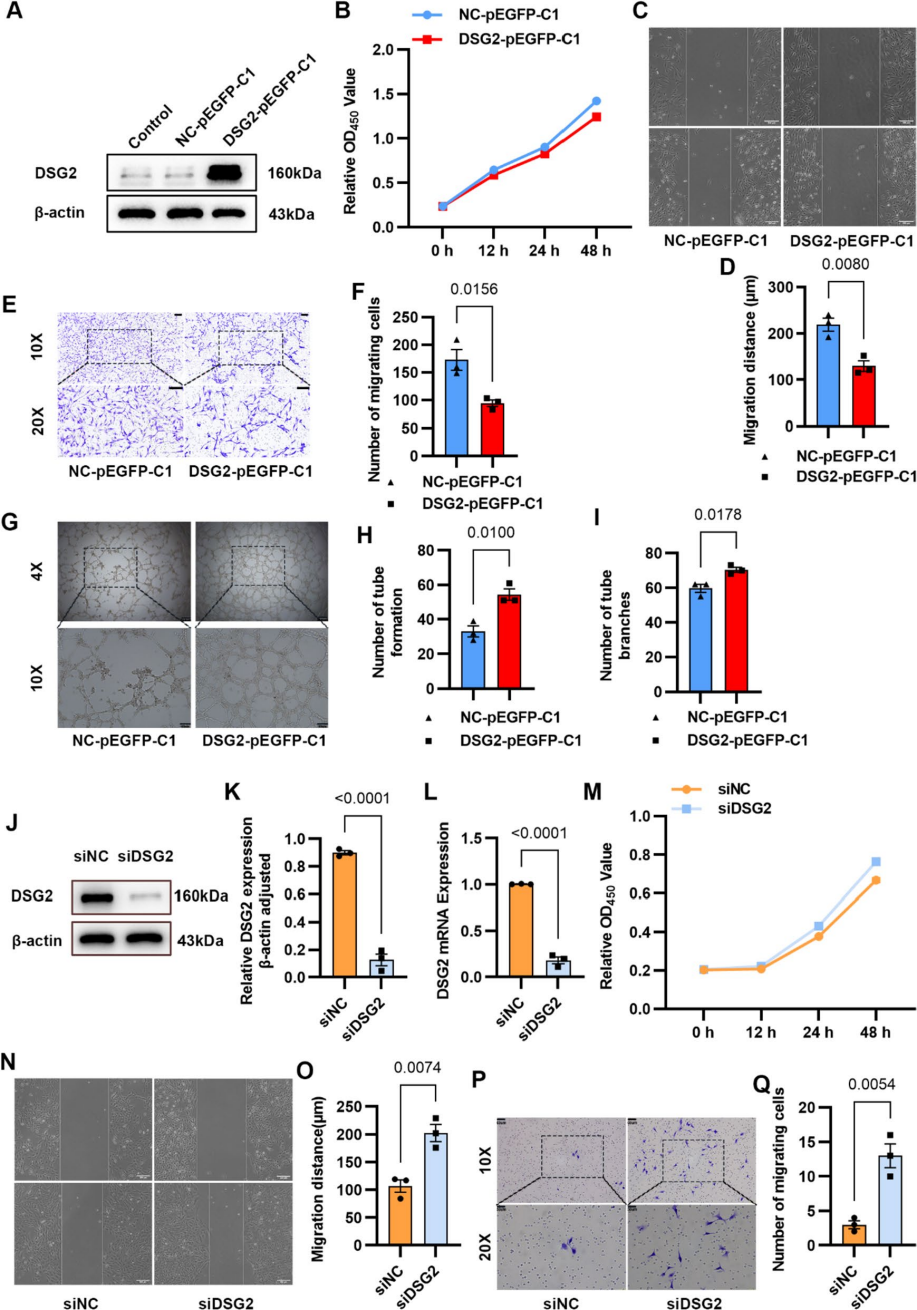
DSG2 inhibited proliferation and migration and promoted tube formation in endothelial cells.** A** Overexpression of DSG2 protein in hCMEC/D3 cells for 24 h, the protein of DSG2 was detected by Western blot; **B** the viability of hCMEC/D3 cells was detected by CCK-8 assay; **C**, **D**, **E**, **F** the migration ability of hCMEC/D3 cells was detected by wound healing experiment and Transwell migration experiment. Scale bar: 200 µm and 100 µm; **G**, **H**, **I**: the tube formation ability of hCMEC/D3 cells was detected by the tube formation experiment. Scale bar: 100 µm and 200 µm. **J**, **K**, **L** knockdown of DSG2 protein in HUVECs for 48 h, the protein and mRNA levels of DSG2 were detected by Western blot analysis and qPCR; **M** the proliferation ability of HUVECs after DSG2 knockdown was detected by CCK-8; **N**, **O**, **P**, **Q** wound healing assay and Transwell migration assay were used to detect the migration ability of HUVECs. Scale bar: 200 µm, 60 µm and 30 µm. DSG2, desmoglein-2

To validate the biological role of DSG2 in Moyamoya disease, NC-pEGFP-C1 and DSG2-pEGFP-C1 plasmids were transfected into hCMEC/D3 cells to overexpress DSG2, and siRNA-NC and siRNA-DSG2 were transfected into HUVECs to knockdown DSG2. The results of Western blot showed the DSG2-pEGFP-C1 plasmid was successfully transferred into hCMEC/D3 cells and expressed successfully (Fig. 2A). Overexpression of DSG2 inhibited cell proliferation (Fig. 2B) and inhibited cell migration (Fig. 2C–F) but promoted tube formation in cells, and the tube formation structure was tighter and less prone to collapse (Fig. 2G–I). Western blot and qPCR showed that DSG2 was successfully knockdown in HUVECs (Fig. 2J–L), and knockdown of DSG2 promoted the proliferation (Fig. 2M) and migration (Fig. 2N–Q) of HUVECs. The above results suggested that DSG2 inhibits Moyamoya disease–related endothelial cell proliferation and migration but promotes tube formation.

### Cobalt-Produced Hypoxia Affects DSG2 Levels, Cell Proliferation, and Migration

**Fig. 3:**
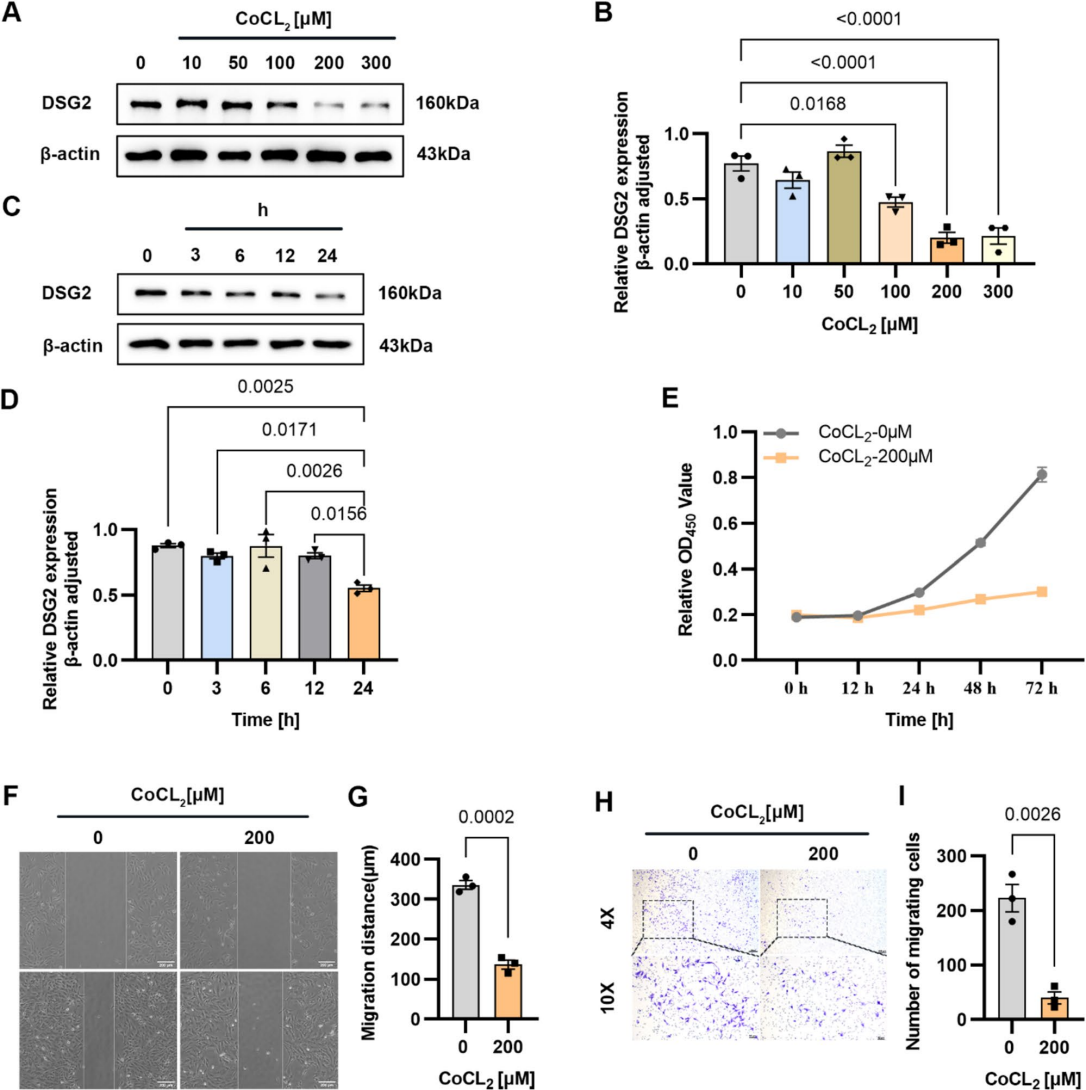
Cobalt-produced hypoxia affects DSG2 levels, cell proliferation, and migration.** A**, **B**, **C**, **D** Western blot was used to detect the protein level of DSG2 in HUVECs stimulated with CoCL_2_; **E** CCK-8 assay was used to detect the cell viability of HUVECs stimulated with 200 µM CoCL_2_ for 24 h; **F**, **G**, **H**, **I** wound healing assay and Transwell migration assay were used to detect the migration ability of HUVECs stimulated with 200 µM CoCL_2_ for 24 h. Scales bar: 200 µm and 60 µm. CoCl_2_, cobalt chloride; DSG2, desmoglein-2

It is well-known that Moyamoya disease is a chronic ischemic cerebrovascular occlusive disease in which there is insufficient oxygen supply to tissues during ischemia. We used CoCL_2_ stimulation of HUVECs as a hypoxia model to verify the effect of hypoxia on DSG2. The results showed that hypoxic by CoCL_2_ inhibited the protein levels of DSG2, and the effect was most obvious at 24 h of 200 µM CoCl_2_ stimulation (Fig. 3A–D). Hypoxia inhibited the proliferation (Fig. 3E) and migration (Fig. 3F–I) of HUVECs. These findings suggest that cobalt-produced downregulation in DSG2 expression was associated with decreased proliferation and migration in HUVECs.

### DSG2 Inhibits Apoptosis of Vascular Endothelial Cells

**Fig. 4:**
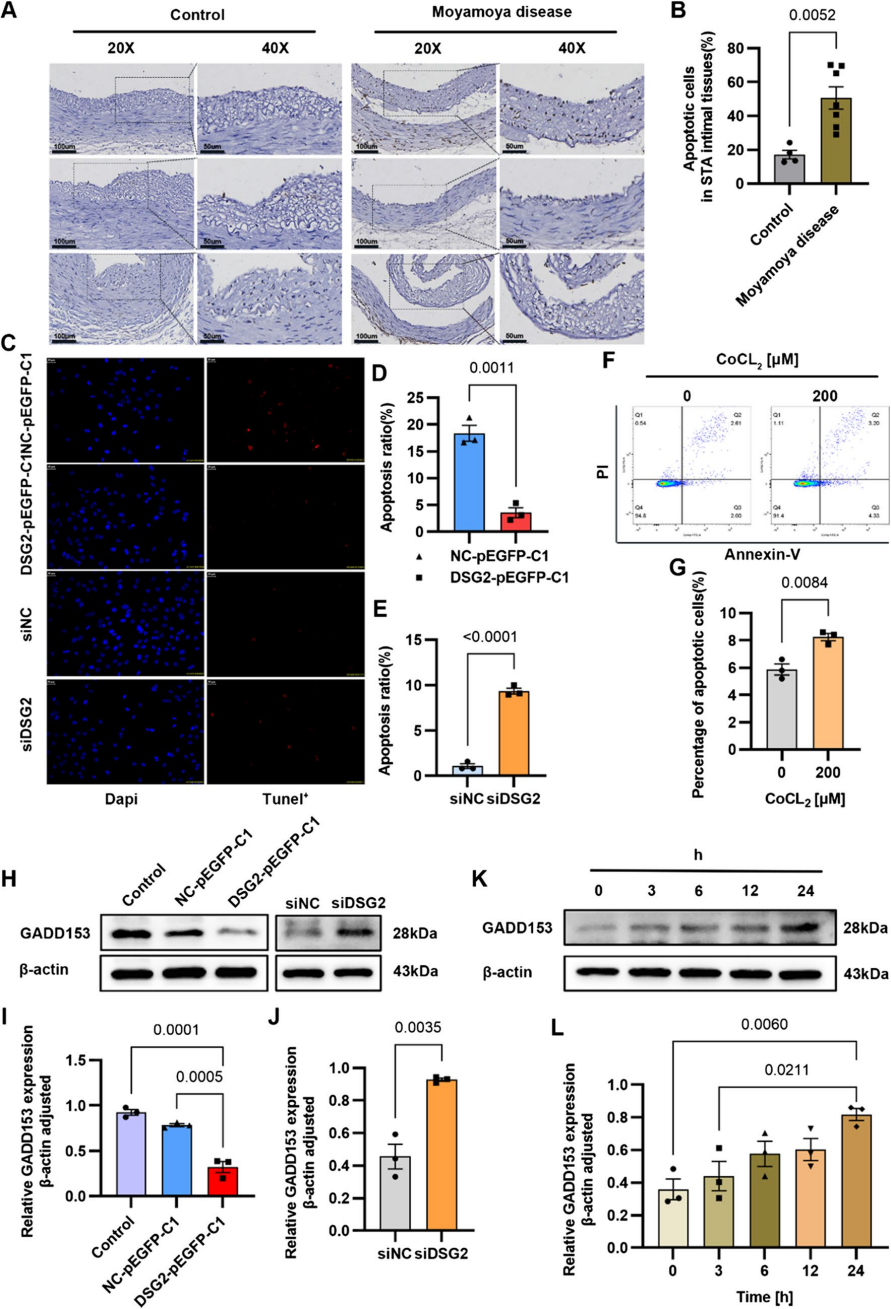
DSG2 inhibits apoptosis of vascular endothelial cells.** A**, **B** Tunel detected apoptosis of STA endothelial cells and intimal tissues in Moyamoya disease and normal controls. Scale bar: 100 µm and 50 µm; **C**, **D**, **E** tunel detected apoptosis of the hCMEC/D3 cells overexpressing DSG2 and HUVECs knockdown DSG2. Scale bar: 30 µm; **F**, **G** Flow cytometry detected apoptosis rates of HUVECs stimulated with 200 µM CoCL_2_ for 24 h; **H**, **K**, **I**, **J**, **L** Western blot detected protein levels of GADD153 in the hCMEC/D3 cells overexpressing DSG2, HUVEC knockdown DSG2 and HUVECs stimulated with 200 µM CoCL2 for 24 h. CoCl_2_, cobalt chloride; DSG2, desmoglein-2; GADD153, pro-apoptotic transcription factor C/EBP-homologous protein (CHOP)

TUNEL assay was performed to detect apoptosis in paraffin sections of STA tissues from Moyamoya disease and normal controls. The results showed that compared with the normal control, the apoptosis rate of STA endothelial cells in the Moyamoya disease group was significantly increased (Fig. 4A, B). To verify the effect of DSG2 on apoptosis, we detected the apoptosis of the hCMEC/D3 cells overexpressing DSG2 and HUVECs knockdown DSG2, and the results of TUNEL showed that the TUNEL-positive cells of DSG2 overexpression group were reduced, and the TUNEL-positive cells of HUVECs knockdown DSG2 was increased (Fig. 4C–E). Next, we detected the apoptosis of HUVECs induced by CoCL_2_ by FITC. Compared with the blank control group, the apoptosis rate of HUVECs with hypoxia was increased (Fig. 4F, G). The Western blot results also showed that overexpression of DSG2 inhibited the expression of the pro-apoptotic transcription factor C/EBP-homologous protein (CHOP)/GADD153, and knockdown DSG2 and hypoxia promoted the expression of GADD153 protein (Fig. 4H–L). These results indicate that DSG2 inhibits the apoptosis of vascular endothelial cells.

### DSG2 Affects PI3K Signaling in Vascular Endothelial Cells

**Fig. 5:**
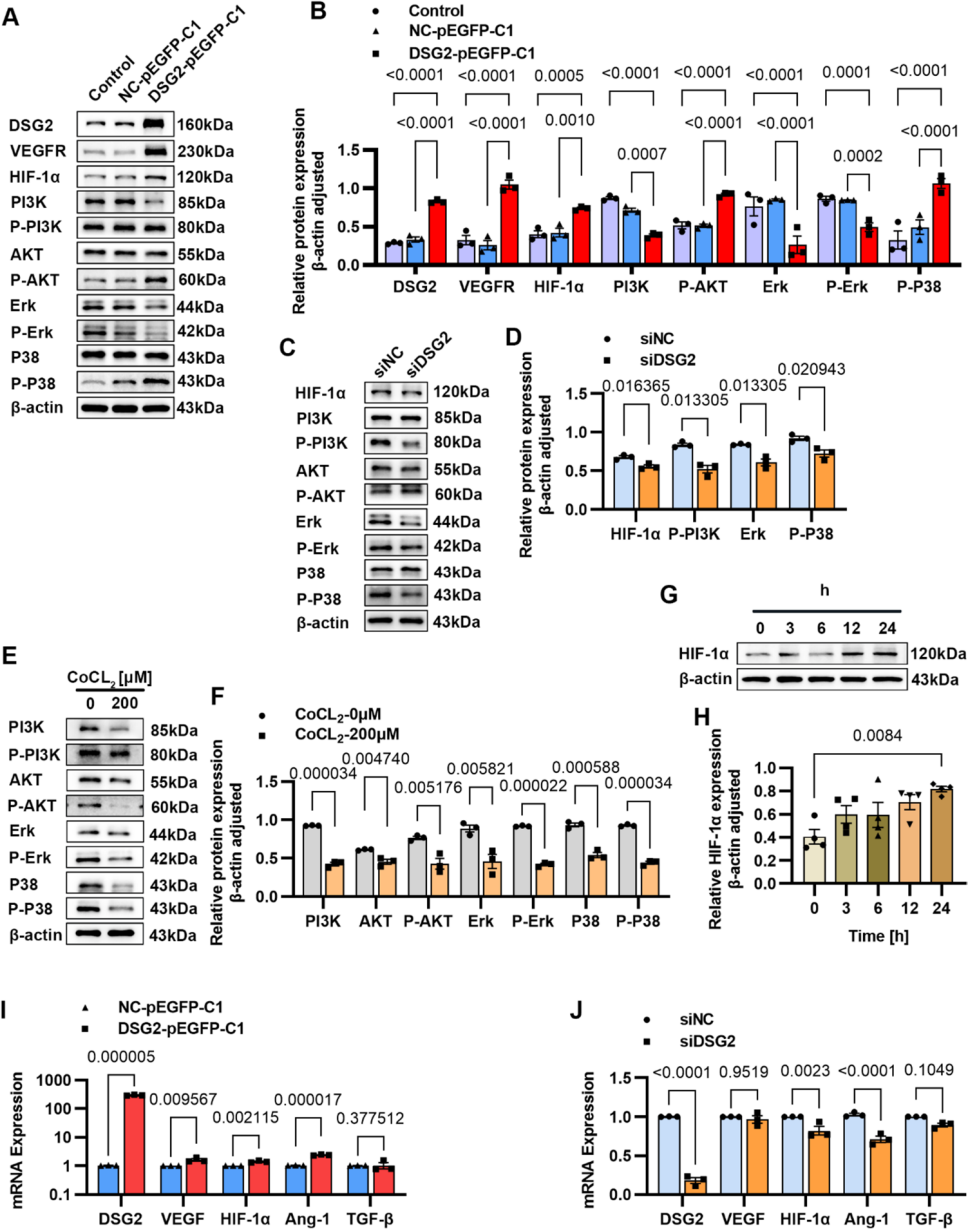
DSG2 affects PI3K signaling in vascular endothelial cells.** A**, **B**, **C**, **D** Western blot was performed to examine the effect of overexpression of DSG2 and knockdown of DSG2 on levels of angiogenesis-related pathway proteins; **E**, **F**, **G**, **H** Western blot to detect the effect of hypoxia on the levels of angiogenesis-related pathway proteins; **I**, **J** QPCR to examine the effect of overexpression of DSG2 and knockdown of DSG2 on transcript levels of angiogenesis-related factors. CoCl_2_, cobalt chloride; DSG2, desmoglein-2; VEGFR, vascular endothelial growth factor receptor; HIF-1α, hypoxia-inducible factor 1-alpha; PI3K, phosphatidylinositol 3-kinase; P-PI3K, phospho-phosphatidylinositol 3-kinase; AKT, protein kinase B; P-AKT, phospho-protein kinase B; ErK, extracellular regulated protein kinases; P-ErK, phospho-extracellular regulated protein kinases; P38, p38 MAPK; P-P38, phospho-p38 MAPK; VEGF, vascular endothelial growth factor; Ang-1, angiopoietin-1 protein; TGF-β, transforming growth factor-β

To verify which way DSG2 inhibits the apoptosis of vascular endothelial cells, we performed Western blot experiments on related pathway proteins, and the results showed that overexpression of DSG2 promoted the expression of VEGFR and HIF-1αproteins, promoted the phosphorylation of AKT and P38, inhibited the expression of PI3K, Erk, and P38 proteins, and inhibited the phosphorylation of Erk (Fig. 5A, B). Knockdown DSG2 inhibited the expression of HIF-1α and Erk proteins and inhibited the phosphorylation of PI3K and P38 (Fig. 5C, D). In CoCL_2_ treatment, the expression of HIF-1α increased in a time-dose, but the expression of proteins such as AKT, and Erk was inhibited, and the phosphorylation of AKT, Erk, and P38 was also inhibited (Fig. 5E–H). In mRNA levels, the qPCR results showed that DSG2 overexpression promoted the expression of angiogenesis-related genes such as VEGF, HIF-1α, and Ang-1 (Fig. 5I), and knockdown DSG2 inhibited the transcription of HIF-1α and Ang-1 (Fig. 5J). These results suggest that DSG2 may function in vascular endothelial cells via PI3K signaling, and DSG2 may disrupt the balance between pro-angiogenic and anti-angiogenic factors.

### MMP-9 Is Involved in DSG2-Mediated Behavior Changes of STA Endothelial Cells in Moyamoya Disease

**Fig. 6:**
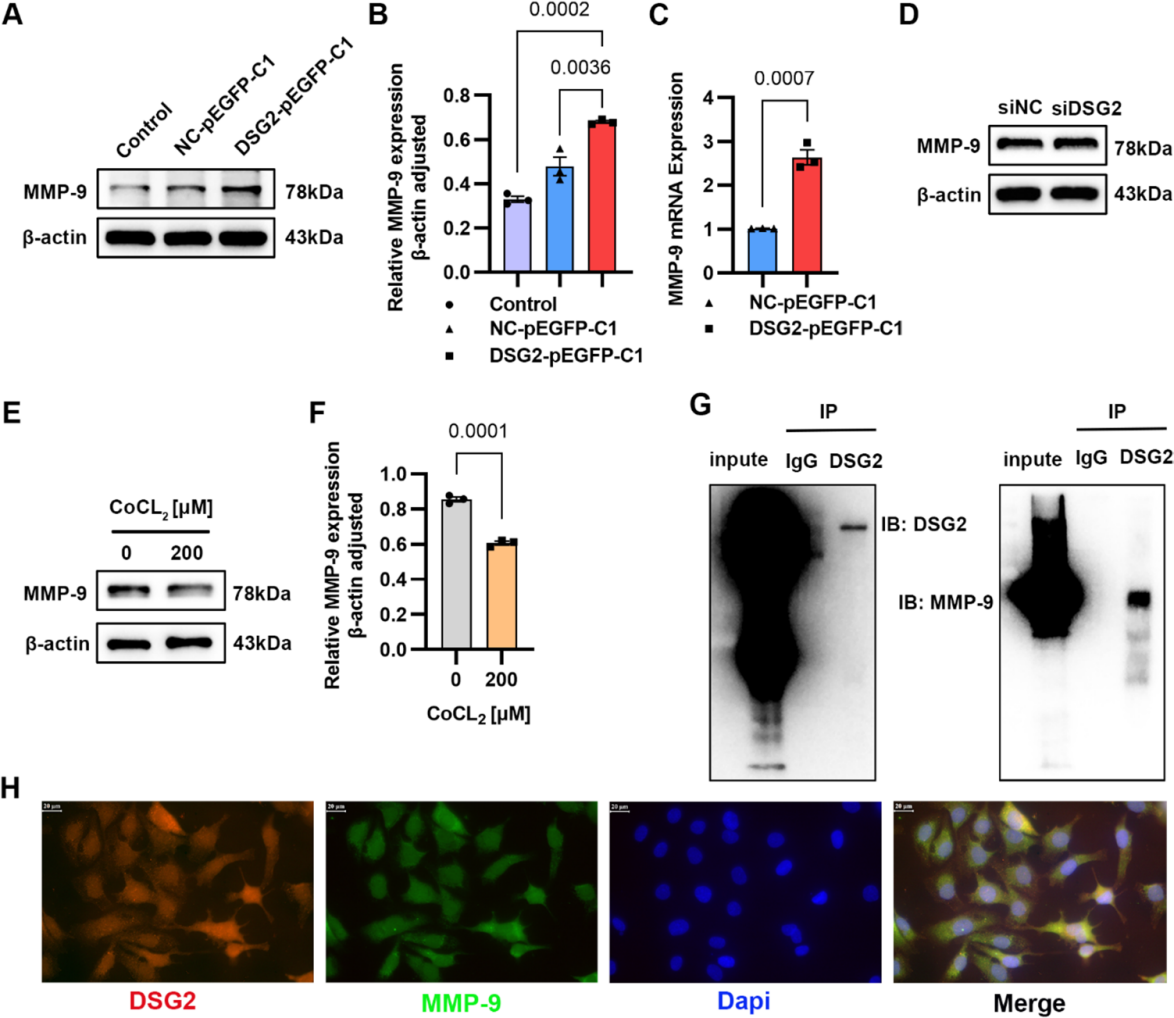
MMP-9 is involved in DSG2-mediated behavior changes of STA endothelial cells in Moyamoya disease. **A**, **B**, **C** Western blot and qPCR were used to detect the effect of overexpression of DSG2 on MMP-9 protein and mRNA levels; **D** Western blot was used to detect the effect of knockdown of DSG2 on MMP-9 protein level; **E**, **F** Western blot was used to detect the protein level of MMP-9 in HUVECs stimulated with 200 µM CoCL_2_ for 24 h; **G** co-immunoprecipitation results showed an interaction between DSG2 and MMP-9; IP, immunoprecipitation; IB, immunoblot. H: immunofluorescence microscopy results showed a co-localization between DSG2 and MMP-9, DSG2 was red and MMP-9 was green. scale bar: 20 µm. CoCl_2_, cobalt chloride; DSG2, desmoglein-2; MMP-9, matrix metallopeptidase 9; Dapi, 4′,6-diamidino-2-phenylindole

We detected the expression of related proteins in DSG2 overexpression and DSG2 low-expression cell lines. Western blot and qPCR results showed that the overexpression of DSG2 promoted the expression of MMP-9 (Fig. 6A–C), but knockdown DSG2 had no effect on the level of MMP-9 protein (Fig. 6D), and hypoxia inhibited the expression of MMP-9 (Fig. 6E, F). Co-immunoprecipitation was used to verify the interaction between proteins, and the results showed that there was an interaction between DSG2 and MMP-9 (Fig. 6G). In addition, immunofluorescence results showed that DSG2 (red) and MMP-9 (green) were co-expressed and localized in endothelial cells (Fig. 6H). These results indicate that there is an interaction between DSG2 and MMP-9 in vascular endothelial cells.

Taken together, these results suggest that DSG2 affects PI3K signaling in vascular endothelial cells, and MMP-9 is involved in DSG2-mediated vascular changes in Moyamoya disease.

## Discussion

The main findings of this study are as follows: (a) DSG2 level decreased in patients with Moyamoya disease, (b) DSG2 affects PI3K signaling in vascular endothelial cells, and (c) MMP-9 is involved in DSG2-mediated vascular changes in Moyamoya disease.

At present, the pathophysiology of Moyamoya disease has not yet been elucidated. From genetic linkage studies and association studies to DNA sequencing, including whole genome sequencing, these technologies continue to achieve new results in the genetic basis of Moyamoya disease [14, 15]. We analyzed the gene expression difference of Moyamoya disease by RNA sequencing and found that the expression of DSG2 decreased significantly in Moyamoya disease as confirmed by Western blot and immunohistochemical validation. The results showed that compared with the normal control, the STA of MMD had the same pathological changes as the intracranial arteries, such as thickening of the intima and irregular internal elastic layer [16, 17].

DSG2 is expressed in endothelial cells and is associated with cell proliferation, migration, invasion, and tube formation [9–12]. In this study, overexpression of DSG2 was found to inhibit the proliferation and migration of the hCMEC/D3 cells but promote tube formation, and knockdown of DSG2 promotes proliferation and migration of HUVECs. Related studies have revealed the association of VEGF, MMP, HIF-1α, and TGF-β with MMD and are closely related to MMD vascular remodeling [18–22]. QPCR also confirmed that DSG2 affects the transcription of various proangiogenic factors such as VEGF, MMP-9, HIF-1α, and Ang-1, and DSG2 may lead to abnormal vascular development by disturbing the balance between proangiogenic and antiangiogenic factors.

Abnormalities in apoptosis are widespread in vascular endothelial cells in Moyamoya disease and apoptosis may play an important role in the development of Moyamoya disease [23]. In this study, we also found abnormalities in endothelial cell apoptosis in the STA of Moyamoya disease, overexpression of DSG2 inhibits apoptosis and inhibits the expression of the pro-apoptotic transcription factor GADD153, DSG2 knockdown promotes apoptosis of HUVECs and enhances the expression of GADD153, a cellular stress–induced transcription factor, which is thought to play a role in the signal transduction from stress endoplasmic reticulum and cerebral ischemic stress to apoptosis, and is closely related to apoptosis [24, 25].

Moyamoya disease is a chronic hypoxic-ischemic cerebrovascular disease. We also investigated the effect of hypoxia on DSG2 levels. In vitro studies showed that the expression of HIF-1α increased with the duration of hypoxia induced by CoCL_2_, and CoCL_2_ hypoxia stimulation significantly inhibited the expression of DSG2, promoted the apoptosis of HUVECs, and promoted the expression of GADD153 at 24 h. However, cobalt-produced downregulation in DSG2 expression was associated with decreased proliferation and migration in HUVECs, which may be associated with hypoxic stimulation by CoCL_2_ [26].

Previous studies have shown that DSG2 can regulate the biological behavior of cells through a variety of signaling pathways such as EGFR, PI3K/Akt, ERK, and JAK/STAT pathways [27, 28]. To verify the mechanism of DSG2 in the biological behavior of vascular endothelial cells, we performed Western blot experiments on related pathway proteins. The results showed that the expression level of PI3K signaling-related proteins changed with the change of DSG2 expression, and hypoxia induced the change of PI3K signaling-related protein level, indicating that DSG2 may affect the biological behavior of vascular endothelial cells through PI3K signaling, thereby promoting the progression of Moyamoya disease.

Plasma and serum MMP-9 levels are elevated in Moyamoya disease, and MMP-9 regulates angiogenic and angiogenic processes by targeting collagen IV and other extracellular matrices, leading to pathological angiogenesis in Moyamoya disease [18, 29]. MMP-9 also promotes angiogenesis by releasing and activating growth factors and cytokines but also inhibits angiogenesis by generating angiogenic inhibitors and split angiogenic factors [30, 31]. Research show [32, 33] that MMP-9 promotes the shedding of the extracellular domain of DSG2 in epithelial cells, and the shed fragments promote cell proliferation and migration, so we speculate that there may be a role between DSG2 and MMP-9 in endothelial cells. We found that overexpression of DSG2 promoted the transcription and translation of MMP-9, and Co-IP confirmed that DSG2 indeed interacted with MMP-9. DSG2 may interact with MMP-9 in Moyamoya disease vascular endothelial cells, causing impaired skeleton regulation in vascular endothelial cells in Moyamoya disease, which in turn leads to pathological vascular remodeling in Moyamoya disease and the interaction between the two may serve as a target for future treatment, which in turn inhibits the further development of Moyamoya disease.

## Conclusions

**Fig. 7:**
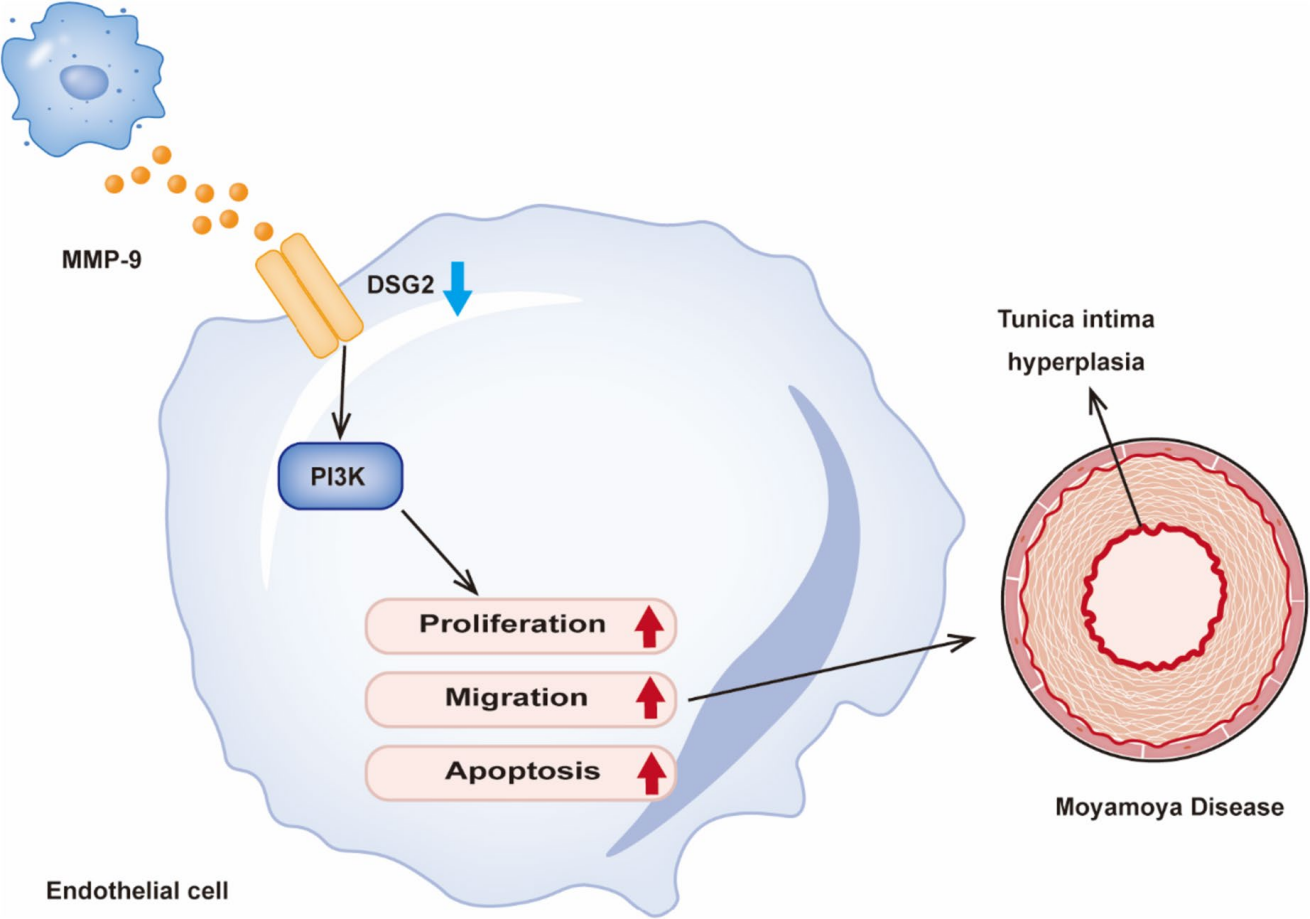
Schematic diagram of the possible regulatory effect of DSG2 in endothelial cells. DSG2 regulates endothelial cell proliferation, migration, tubule formation, and apoptosis by interacting with MMP9 and activating the PI3K signaling pathway, thereby contributing to Moyamoya disease vascular remodeling. MMP-9, matrix metallopeptidase 9; DSG2, desmoglein-2; PI3K, phosphatidylinositol 3-kinase

DSG2 affects PI3K signaling in vascular endothelial cells, and MMP-9 is involved in DSG2-mediated vascular changes in Moyamoya disease to balance pro-angiogenic and anti-angiogenic factors, thus resulting in abnormalities in endothelial cell proliferation, migration, tube formation, and apoptosis, which are associated with Moyamoya disease vascular remodeling (Fig. 7).

### Supplementary Information

Below is the link to the electronic supplementary material.

Supplementary file1 (TIF 18877 KB)

Supplementary file2 (JPG 90 KB)

Supplementary file3 (PDF 56 KB)

## Data Availability

The datasets generated during and/or analyzed during the current study are available from the corresponding author upon reasonable request.
