# Correction: Desmoglein-2 Affects Vascular Function in Moyamoya Disease by Interacting with MMP-9 and Influencing PI3K Signaling

**DOI:** 10.1007/s12035-024-04152-1

**Published:** 2024-04-15

**Authors:** Ajun Wang, Nan Li, Nan Zhang, Jian Liu, Tao Yang, Dongxue Li, Changwen Li, Rui Li, Tongcui Jiang, Chengyu Xia

**Affiliations:** 1 Department of Neurosurgery, The First Affiliated Hospital of USTC, Division of Life Science and Medicine, University of Science and Technology of China, 17 Lujiang Road, Hefei, Anhui Province China; 2 Department of Neurosurgery, Anhui Provincial Hospital, Affiliated to Anhui Medical University, Hefei, China; 3 School of Basic Medical Sciences, Anhui Medical University, 81 Meishan Road, Hefei, Anhui Province China

**Correction: Molecular Neurobiology**

10.1007/s12035-024-04010-0

The original version of this article unfortunately contained error in Figure 4F.

In Figure 4F, CoCL2-0um and CoCL2-200um flow apoptosis picture position is reversed. Everything else is correct and we have made a new Figure 4 below.

There were the below errors when this article first published online.
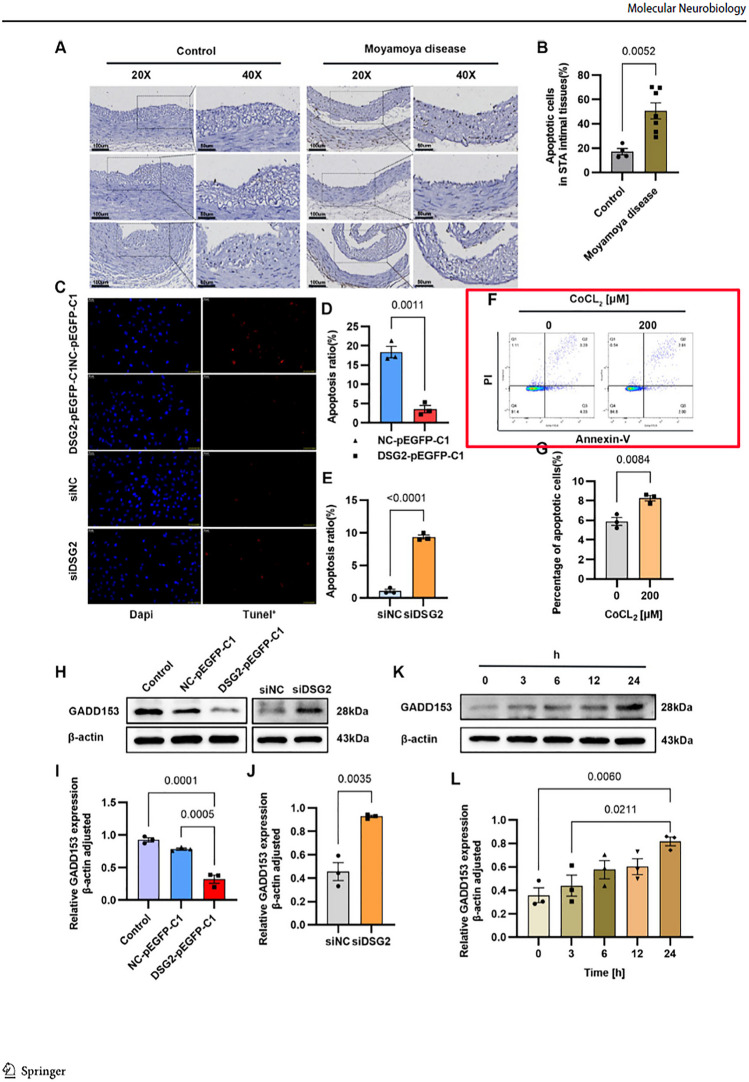


Corrected Figure 4
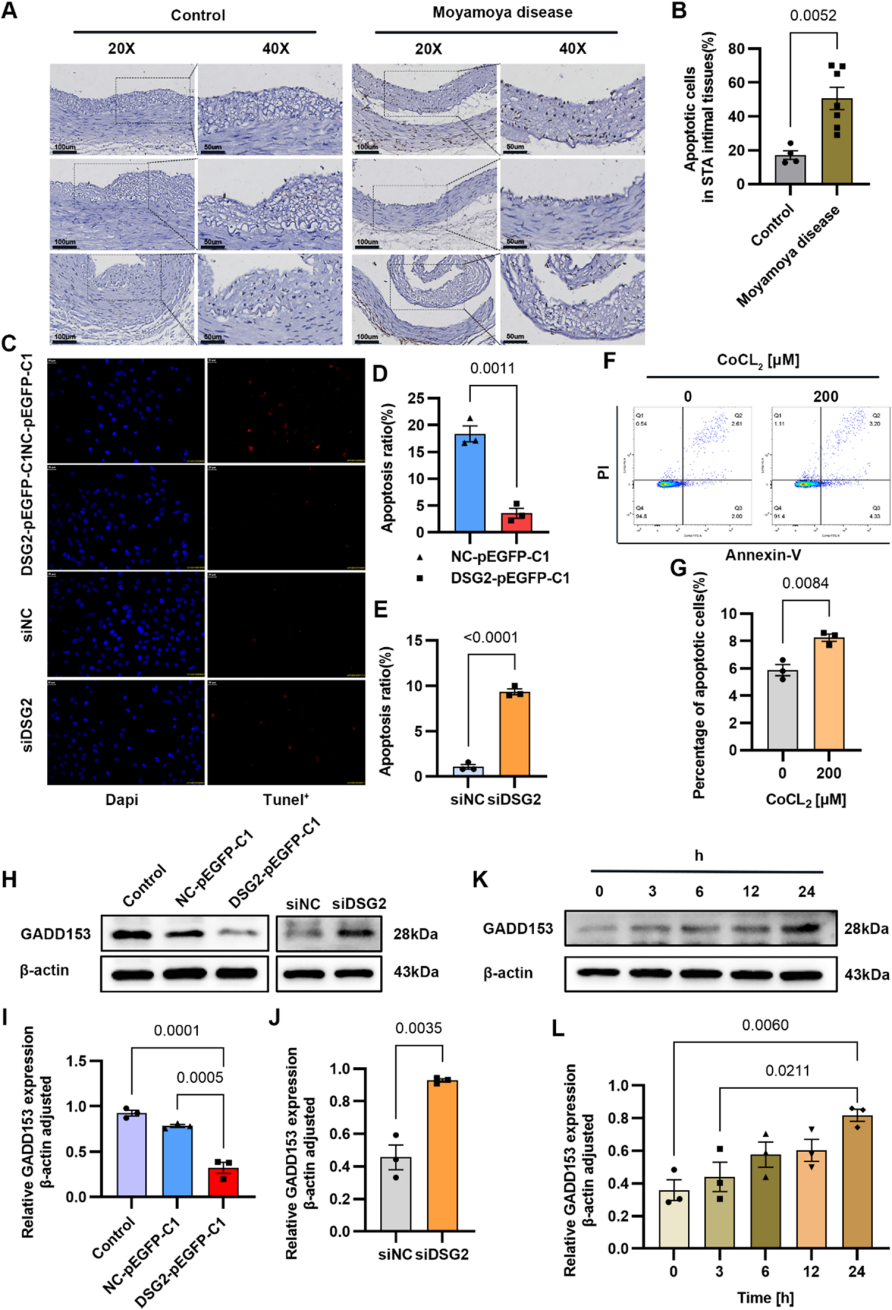


The original article has been corrected.

