# Supplementary figures and images for: Desmoglein-2 Affects Vascular Function in Moyamoya Disease by Interacting with MMP-9 and Influencing PI3K Signaling
Source: Mol Neurobiol. 2024 Feb 7;61(9):6539–52. doi: 10.1007/s12035-024-04010-0 (PMC11339177; doi:10.1007/s12035-024-04010-0)

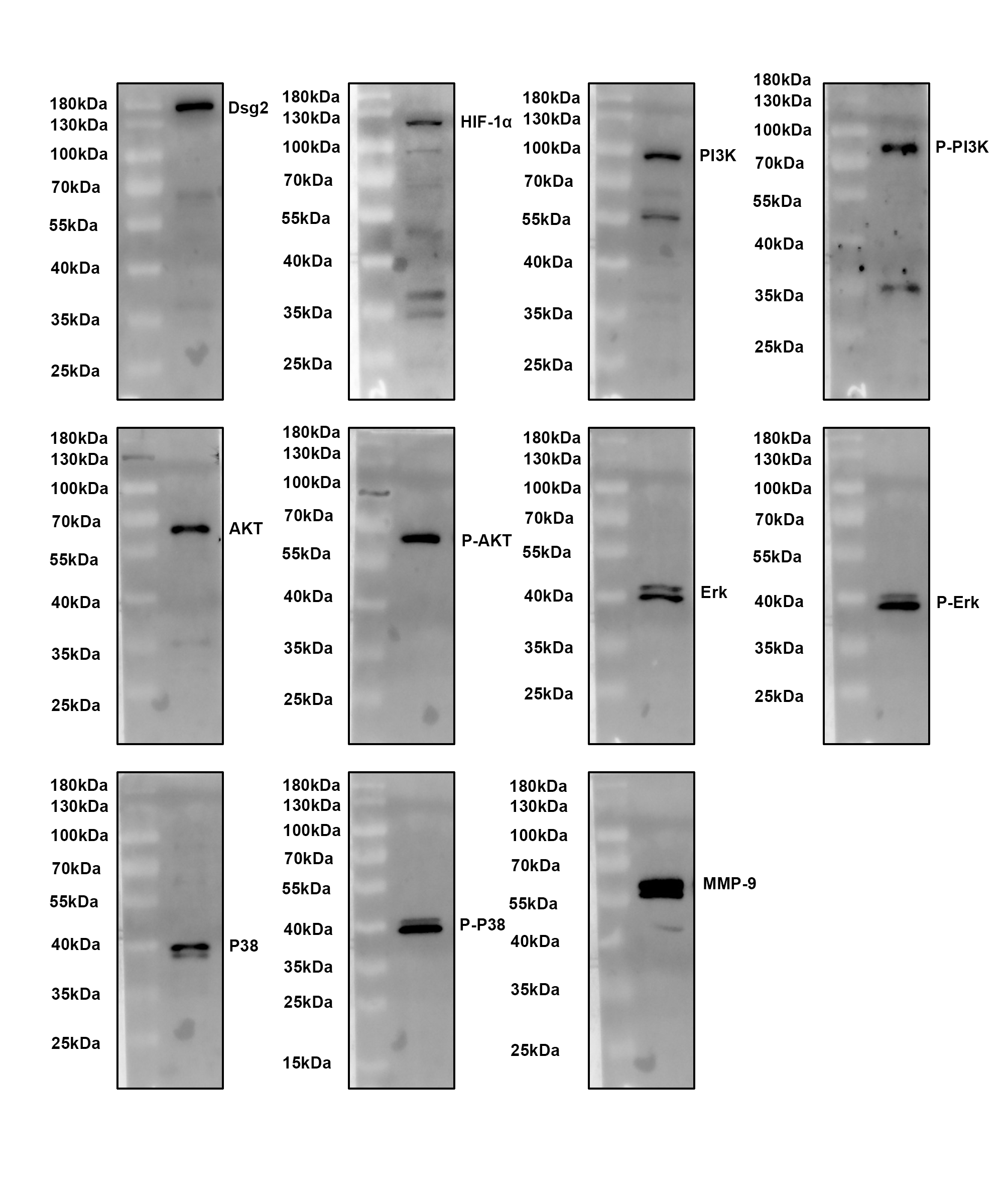

Supplement: Supplementary file 1 — Supplementary file1 (TIF 18877 KB) [file 12035_2024_4010_MOESM1_ESM.tif]

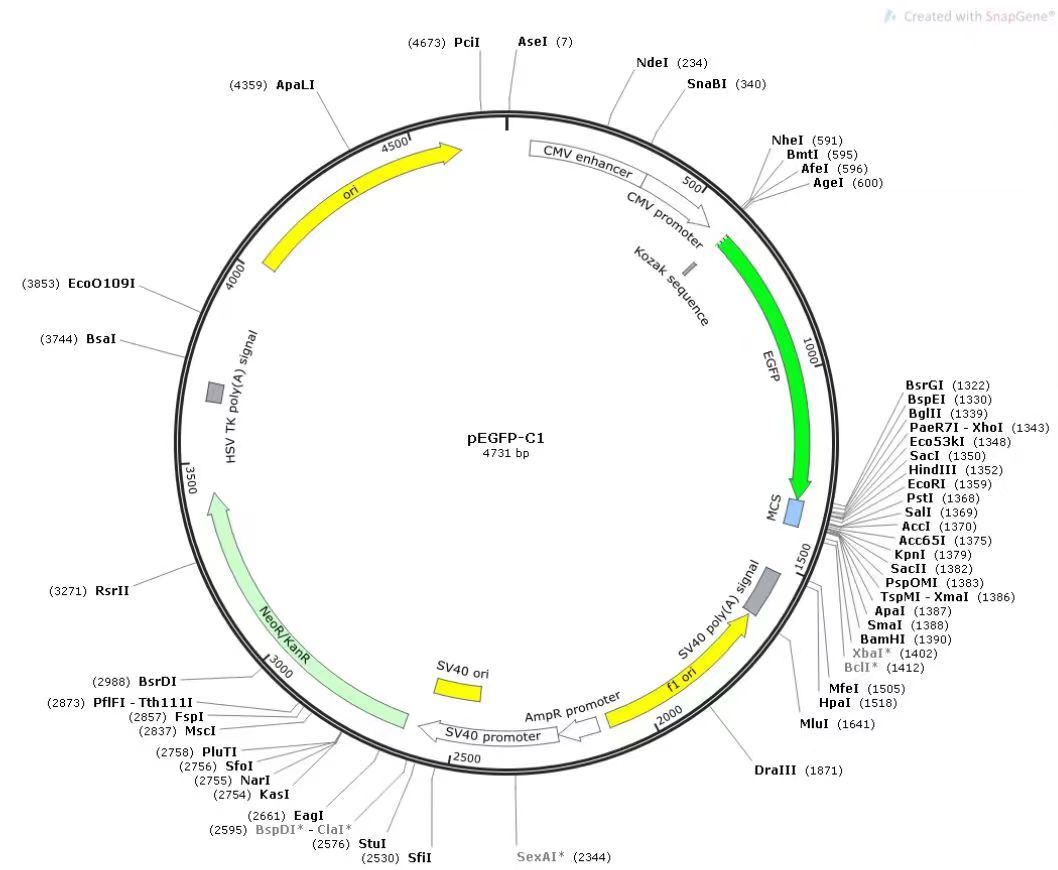

Supplement: Supplementary file 2 — Supplementary file2 (JPG 90 KB) [file 12035_2024_4010_MOESM2_ESM.jpg]
