# Supplementary material for: Desmoglein-2 Affects Vascular Function in Moyamoya Disease by Interacting with MMP-9 and Influencing PI3K Signaling
Source: Mol Neurobiol. 2024 Feb 7;61(9):6539–52. doi: 10.1007/s12035-024-04010-0 (PMC11339177; doi:10.1007/s12035-024-04010-0)
Supplement: Supplementary file 3 — Supplementary file3 (PDF 56 KB) [file 12035_2024_4010_MOESM3_ESM.pdf]

10 20 30 40 50 60 70 80 90 100 110

GGACTCAGATCTCGAGCTCAAGCTTCGAATTCTGCAGTCGACATGGCGCGGAGCCCGGGACGCGCGTACGCCCTGCTGCTTCTCCTGATCTGCTTTAACGTTGGAAGTGG

E02-PD64524-C5276HH080-4-PEGFP-C-5.ab1(1>755) → GGACTCAGATCTCGAGCTCAAGCTTCGAATTCTGCAGTCGACATGGCGCGGAGCCCGGGACGCGCGTACGCCCTGCTGCTTCTCCTGATCTGCTTTAACGTTGGAAGTGG
C5276HH080-4.seq(1>3369) → GTCGACATGGCGCGGAGCCCGGGACGCGCGTACGCCCTGCTGCTTCTCCTGATCTGCTTTAACGTTGGAAGTGG

120 130 140 150 160 170 180 190 200 210 220

ACTTCACTTACAGGTCTTAAGCACAAGAAATGAAAATAAGCTGCTTCCTAAACATCCTCATTTAGTGCGGCAAAAGCGCGCCTGGATCACCGCCCCCGTGGCTCTTCGGG

E02-PD64524-C5276HH080-4-PEGFP-C-5.ab1(1>755) → ACTTCACTTACAGGTCTTAAGCACAAGAAATGAAAATAAGCTGCTTCCTAAACATCCTCATTTAGTGCGGCAAAAGCGCGCCTGGATCACCGCCCCCGTGGCTCTTCGGG
C5276HH080-4.seq(1>3369) → ACTTCACTTACAGGTCTTAAGCACAAGAAATGAAAATAAGCTGCTTCCTAAACATCCTCATTTAGTGCGGCAAAAGCGCGCCTGGATCACCGCCCCCGTGGCTCTTCGGG

230 240 250 260 270 280 290 300 310 320 330

AGGGAGAGGATCTGTCCAAGAAGAATCCAATTGCCAAGATACATTCTGATCTTGCAGAAGAAAGAGGACTCAAAATTACTTACAAATACACTGGAAAAGGGATTACAGAG

E02-PD64524-C5276HH080-4-PEGFP-C-5.ab1(1>755) → AGGGAGAGGATCTGTCCAAGAAGAATCCAATTGCCAAGATACATTCTGATCTTGCAGAAGAAAGAGGACTCAAAATTACTTACAAATACACTGGAAAAGGGATTACAGAG
C5276HH080-4.seq(1>3369) → AGGGAGAGGATCTGTCCAAGAAGAATCCAATTGCCAAGATACATTCTGATCTTGCAGAAGAAAGAGGACTCAAAATTACTTACAAATACACTGGAAAAGGGATTACAGAG

340 350 360 370 380 390 400 410 420 430 440

CCACCTTTTGGTATATTTGTCTTTAACAAAGATACTGGAGAACTGAATGTTACCAGCATTCTTGATCGAGAAGAAACACCATTTTTTCTGCTAACAGGTTACGCTTTGGA

E02-PD64524-C5276HH080-4-PEGFP-C-5.ab1(1>755) → CCACCTTTTGGTATATTTGTCTTTAACAAAGATACTGGAGAACTGAATGTTACCAGCATTCTTGATCGAGAAGAAACACCATTTTTTCTGCTAACAGGTTACGCTTTGGA
C5276HH080-4.seq(1>3369) → CCACCTTTTGGTATATTTGTCTTTAACAAAGATACTGGAGAACTGAATGTTACCAGCATTCTTGATCGAGAAGAAACACCATTTTTTCTGCTAACAGGTTACGCTTTGGA

450 460 470 480 490 500 510 520 530 540 550

TGCAAGAGGAAACAATGTAGAGAAACCCTTAGAGCTACGCATTAAGGTTCTTGATATCAATGACAACGAACCAGTGTTCACACAGGATGTCTTTGTTGGGTCTGTTGAAG

E02-PD64524-C5276HH080-4-PEGFP-C-5.ab1(1>755) → TGCAAGAGGAAACAATGTAGAGAAACCCTTAGAGCTACGCATTAAGGTTCTTGATATCAATGACAACGAACCAGTGTTCACACAGGATGTCTTTGTTGGGTCTGTTGAAG
C5276HH080-4.seq(1>3369) → TGCAAGAGGAAACAATGTAGAGAAACCCTTAGAGCTACGCATTAAGGTTCTTGATATCAATGACAACGAACCAGTGTTCACACAGGATGTCTTTGTTGGGTCTGTTGAAG

560 570 580 590 600 610 620 630 640 650 660

AGTTGAGTGCAGCACATACTCTTGTGATGAAAATCAATGCAACAGATGCAGATGAGCCCAATACCCTGAATTCGAAAATTTCCTATAGAATCGTATCTCTGGAGCCTGCT

E02-PD64524-C5276HH080-4-PEGFP-C-5.ab1(1>755) → AGTTGAGTGCAGCACATACTCTTGTGATGAAAATCAATGCAACAGATGCAGATGAGCCCAATACCCTGAATTCGAAAATTTCCTATAGAATCGTATCTCTGGAGCCTGCT
C5276HH080-4.seq(1>3369) → AGTTGAGTGCAGCACATACTCTTGTGATGAAAATCAATGCAACAGATGCAGATGAGCCCAATACCCTGAATTCGAAAATTTCCTATAGAATCGTATCTCTGGAGCCTGCT

670 680 690 700 710 720 730 740 750 760 770

TATCCTCCAGTGTTCTACCTAAATAAAGATACAGGAGAGATTTATACAACCAGTGTTACCTTGGACAGAGAGGAACACAGCAGCTACACTTTGACAGTAGAAGCAAGAGA

E02-PD64524-C5276HH080-4-PEGFP-C-5.ab1(1>755) → TATCCTCCAGTGTTCTACCTAAATAAAGATACAGGAGAGATTTATACAACCAGTGTTACCTTGGACAGAGAGGAACACAGCAGCTACACTTTGAC
C5276HH080-4.seq(1>3369) → TATCCTCCAGTGTTCTACCTAAATAAAGATACAGGAGAGATTTATACAACCAGTGTTACCTTGGACAGAGAGGAACACAGCAGCTACACTTTGACAGTAGAAGCAAGAGA
E03-PD64524-C5276HH080-4-2-SEQ1FNP.ab1(1>753) → TACAACCAGTGTTACCTTGGACAGAGAGGAACACAGCAGCTACACTTTGACAGTAGAAGCAAGAGA

780 790 800 810 820 830 840 850 860 870 880

TGGCAATGGAGAAGTTACAGACAAACCTGTAAAACAAGCTCAAGTTCAGATTCGTATTTTGGATGTCAATGACAATATACCTGTAGTAGAAAATAAAGTGCTTGAAGGGA

C5276HH080-4.seq(1>3369) → TGGCAATGGAGAAGTTACAGACAAACCTGTAAAACAAGCTCAAGTTCAGATTCGTATTTTGGATGTCAATGACAATATACCTGTAGTAGAAAATAAAGTGCTTGAAGGGA
E03-PD64524-C5276HH080-4-2-SEQ1FNP.ab1(1>753) → TGGCAATGGAGAAGTTACAGACAAACCTGTAAAACAAGCTCAAGTTCAGATTCGTATTTTGGATGTCAATGACAATATACCTGTAGTAGAAAATAAAGTGCTTGAAGGGA

```
                                                        890       900       910       920       930       940       950       960       970       980       990
                                                  TGGTTGAAGAAAATCAAGTCAACGTAGAAGTTACGCGCATAAAAGTGTTCGATGCAGATGAAATAGGTTCTGATAATTGGCTGGCAAATTTTACATTTGCATCAGGAAAT
C5276HH080-4.seq(1>3369)                       →  TGGTTGAAGAAAATCAAGTCAACGTAGAAGTTACGCGCATAAAAGTGTTCGATGCAGATGAAATAGGTTCTGATAATTGGCTGGCAAATTTTACATTTGCATCAGGAAAT
E03-PD64524-C5276HH080-4-2-SEQ1FNP.ab1(1>753)  →  TGGTTGAAGAAAATCAAGTCAACGTAGAAGTTACGCGCATAAAAGTGTTCGATGCAGATGAAATAGGTTCTGATAATTGGCTGGCAAATTTTACATTTGCATCAGGAAAT

                                                       1000      1010      1020      1030      1040      1050      1060      1070      1080      1090      1100
                                                  GAAGGAGGTTATTTCCACATAGAAACAGATGCTCAAACTAACGAAGGAATTGTGACCCTTATTAAGGAAGTAGATTATGAAGAAATGAAGAATCTTGACTTCAGTGTTAT
C5276HH080-4.seq(1>3369)                       →  GAAGGAGGTTATTTCCACATAGAAACAGATGCTCAAACTAACGAAGGAATTGTGACCCTTATTAAGGAAGTAGATTATGAAGAAATGAAGAATCTTGACTTCAGTGTTAT
E03-PD64524-C5276HH080-4-2-SEQ1FNP.ab1(1>753)  →  GAAGGAGGTTATTTCCACATAGAAACAGATGCTCAAACTAACGAAGGAATTGTGACCCTTATTAAGGAAGTAGATTATGAAGAAATGAAGAATCTTGACTTCAGTGTTAT

                                                       1110      1120      1130      1140      1150      1160      1170      1180      1190      1200      1210
                                                  TGTCGCTAATAAAGCAGCTTTTCACAAGTCGATTAGGAGTAAATACAAGCCTACACCCATTCCCATCAAGGTCAAAGTGAAAAATGTGAAAGAAGGCATTCATTTTAAAA
C5276HH080-4.seq(1>3369)                       →  TGTCGCTAATAAAGCAGCTTTTCACAAGTCGATTAGGAGTAAATACAAGCCTACACCCATTCCCATCAAGGTCAAAGTGAAAAATGTGAAAGAAGGCATTCATTTTAAAA
E03-PD64524-C5276HH080-4-2-SEQ1FNP.ab1(1>753)  →  TGTCGCTAATAAAGCAGCTTTTCACAAGTCGATTAGGAGTAAATACAAGCCTACACCCATTCCCATCAAGGTCAAAGTGAAAAATGTGAAAGAAGGCATTCATTTTAAAA

                                                       1220      1230      1240      1250      1260      1270      1280      1290      1300      1310      1320
                                                  GCAGCGTCATCTCAATTTATGTTAGCGAGAGCATGGATAGATCAAGCAAAGGCCAAATAATTGGAAATTTTCAAGCTTTTGATGAGGACACTGGACTACCAGCCCATGCA
C5276HH080-4.seq(1>3369)                       →  GCAGCGTCATCTCAATTTATGTTAGCGAGAGCATGGATAGATCAAGCAAAGGCCAAATAATTGGAAATTTTCAAGCTTTTGATGAGGACACTGGACTACCAGCCCATGCA
E03-PD64524-C5276HH080-4-2-SEQ1FNP.ab1(1>753)  →  GCAGCGTCATCTCAATTTATGTTAGCGAGAGCATGGATAGATCAAGCAAAGGCCAAATAATTGGAAATTTTCAAGCTTTTGATGAGGACACTGGACTACCAGCCCATGCA

                                                       1330      1340      1350      1360      1370      1380      1390      1400      1410      1420      1430
                                                  AGATATGTAAAATTAGAAGATAGAGATAATTGGATCTCTGTGGATTCTGTCACATCTGAAATTAAACTTGCAAAACTTCCTGATTTTGAATCTAGATATGTTCAAAATGG
C5276HH080-4.seq(1>3369)                       →  AGATATGTAAAATTAGAAGATAGAGATAATTGGATCTCTGTGGATTCTGTCACATCTGAAATTAAACTTGCAAAACTTCCTGATTTTGAATCTAGATATGTTCAAAATGG
E03-PD64524-C5276HH080-4-2-SEQ1FNP.ab1(1>753)  →  AGATATGTAAAATTAGAAGATAGAGATAATTGGATCTCTGTGGATTCTGTCACATCTGAAATTAAACTTGCAAAACTTCCTGATTTTGAATCTAGATATGTTCAAAATGG
E04-PD64524-C5276HH080-4-2-SEQ2FNP.ab1(1>762)  →                                                              CACATCTGAAATTAAACTTGCAAAACTTCCTGATTTTGAATCTAGATATGTTCAAAATGG

                                                       1440      1450      1460      1470      1480      1490      1500      1510      1520      1530      1540
                                                  CACATACACTGTAAAGATTGTGGCCATATCAGAAGATTATCCTAGAAAAACCATCACTGGCACAGTCCTTATCAATGTTGAAGACATCAACGACAACTGTCCCACACTGA
C5276HH080-4.seq(1>3369)                       →  CACATACACTGTAAAGATTGTGGCCATATCAGAAGATTATCCTAGAAAAACCATCACTGGCACAGTCCTTATCAATGTTGAAGACATCAACGACAACTGTCCCACACTGA
E03-PD64524-C5276HH080-4-2-SEQ1FNP.ab1(1>753)  →  CACATACACTGTAAAGATTGTGGCCAT
E04-PD64524-C5276HH080-4-2-SEQ2FNP.ab1(1>762)  →  CACATACACTGTAAAGATTGTGGCCATATCAGAAGATTATCCTAGAAAAACCATCACTGGCACAGTCCTTATCAATGTTGAAGACATCAACGACAACTGTCCCACACTGA

                                                       1550      1560      1570      1580      1590      1600      1610      1620      1630      1640      1650
                                                  TAGAGCCTGTGCAGACAATCTGTCACGATGCAGAGTATGTGAATGTTACTGCAGAGGACCTGGATGGACACCCAAACAGTGGCCCTTTCAGTTTCTCCGTCATTGACAAA
C5276HH080-4.seq(1>3369)                       →  TAGAGCCTGTGCAGACAATCTGTCACGATGCAGAGTATGTGAATGTTACTGCAGAGGACCTGGATGGACACCCAAACAGTGGCCCTTTCAGTTTCTCCGTCATTGACAAA
E04-PD64524-C5276HH080-4-2-SEQ2FNP.ab1(1>762)  →  TAGAGCCTGTGCAGACAATCTGTCACGATGCAGAGTATGTGAATGTTACTGCAGAGGACCTGGATGGACACCCAAACAGTGGCCCTTTCAGTTTCTCCGTCATTGACAAA

                                                       1660      1670      1680      1690      1700      1710      1720      1730      1740      1750      1760
                                                  CCACCTGGCATGGCAGAAAAATGGAAAATAGCACGCCAAGAAAGTACCAGTGTGCTGCTGCAACAAAGTGAGAAAAAGCTTGGGAGAAGTGAAATTCAGTTCCTGATTTC
C5276HH080-4.seq(1>3369)                       →  CCACCTGGCATGGCAGAAAAATGGAAAATAGCACGCCAAGAAAGTACCAGTGTGCTGCTGCAACAAAGTGAGAAAAAGCTTGGGAGAAGTGAAATTCAGTTCCTGATTTC
E04-PD64524-C5276HH080-4-2-SEQ2FNP.ab1(1>762)  →  CCACCTGGCATGGCAGAAAAATGGAAAATAGCACGCCAAGAAAGTACCAGTGTGCTGCTGCAACAAAGTGAGAAAAAGCTTGGGAGAAGTGAAATTCAGTTCCTGATTTC
```

```
                                                              1770      1780      1790      1800      1810      1820      1830      1840      1850      1860      1870
                                                              AGACAATCAGGGTTTTAGTTGTCCTGAAAAGCAGGTCCTTACACTCACAGTTTGTGAGTGTCTGCATGGCAGCGGCTGCAGGGAAGCACAGCATGACTCCTATGTGGGCC
C5276HH080-4.seq(1>3369)                                   →  AGACAATCAGGGTTTTAGTTGTCCTGAAAAGCAGGTCCTTACACTCACAGTTTGTGAGTGTCTGCATGGCAGCGGCTGCAGGGAAGCACAGCATGACTCCTATGTGGGCC
E04-PD64524-C5276HH080-4-2-SEQ2FNP.ab1(1>762)              →  AGACAATCAGGGTTTTAGTTGTCCTGAAAAGCAGGTCCTTACACTCACAGTTTGTGAGTGTCTGCATGGCAGCGGCTGCAGGGAAGCACAGCATGACTCCTATGTGGGCC

                                                              1880      1890      1900      1910      1920      1930      1940      1950      1960      1970      1980
                                                              TGGGACCCGCAGCAATTGCGCTCATGATTTTGGCCTTTCTGCTCCTGCTATTGGTACCACTTTTACTGCTGATGTGCCATTGCGGAAAGGGCGCCAAAGGCTTTACCCCC
C5276HH080-4.seq(1>3369)                                   →  TGGGACCCGCAGCAATTGCGCTCATGATTTTGGCCTTTCTGCTCCTGCTATTGGTACCACTTTTACTGCTGATGTGCCATTGCGGAAAGGGCGCCAAAGGCTTTACCCCC
E04-PD64524-C5276HH080-4-2-SEQ2FNP.ab1(1>762)              →  TGGGACCCGCAGCAATTGCGCTCATGATTTTGGCCTTTCTGCTCCTGCTATTGGTACCACTTTTACTGCTGATGTGCCATTGCGGAAAGGGCGCCAAAGGCTTTACCCCC

                                                              1990      2000      2010      2020      2030      2040      2050      2060      2070      2080      2090
                                                              ATACCTGGCACCATAGAGATGCTGCATCCTTGGAATAATGAAGGAGCACCACCTGAAGACAAGGTGGTGCCATCATTTCTGCCAGTGGATCAAGGGGGCAGTCTAGTAGG
C5276HH080-4.seq(1>3369)                                   →  ATACCTGGCACCATAGAGATGCTGCATCCTTGGAATAATGAAGGAGCACCACCTGAAGACAAGGTGGTGCCATCATTTCTGCCAGTGGATCAAGGGGGCAGTCTAGTAGG
E04-PD64524-C5276HH080-4-2-SEQ2FNP.ab1(1>762)              →  ATACCTGGCACCATAGAGATGCTGCATCCTTGGAATAATGAAGGAGCACCACCTGAAGACAAGGTGGTGCCATCATTTCTGCCAGTGGATCAAGGGGGCAGTCTAGTAGG
E05-PD64524-C5276HH080-4-2-SEQ3FNP.ab1(1>751)              →                                                                                         GATCAAGGGGGCAGTCTAGTAGG

                                                              2100      2110      2120      2130      2140      2150      2160      2170      2180      2190      2200
                                                              AAGAAATGGAGTAGGAGGTATGGCCAAGGAAGCCACGATGAAAGGAAGTAGCTCTGCTTCCATTGTCAAAGGGCAACATGAGATGTCCGAGATGGATGGAAGGTGGGAAG
C5276HH080-4.seq(1>3369)                                   →  AAGAAATGGAGTAGGAGGTATGGCCAAGGAAGCCACGATGAAAGGAAGTAGCTCTGCTTCCATTGTCAAAGGGCAACATGAGATGTCCGAGATGGATGGAAGGTGGGAAG
E04-PD64524-C5276HH080-4-2-SEQ2FNP.ab1(1>762)              →  AAGAAATGGAGTAGGAGGTATGGCCAAGGAAGCCACGATGAA
E05-PD64524-C5276HH080-4-2-SEQ3FNP.ab1(1>751)              →  AAGAAATGGAGTAGGAGGTATGGCCAAGGAAGCCACGATGAAAGGAAGTAGCTCTGCTTCCATTGTCAAAGGGCAACATGAGATGTCCGAGATGGATGGAAGGTGGGAAG

                                                              2210      2220      2230      2240      2250      2260      2270      2280      2290      2300      2310
                                                              AACACAGAAGCCTGCTTTCTGGTAGAGCTACCCAGTTTACAGGGGCCACAGGCGCTATCATGACCACTGAAACCACGAAGACCGCAAGGGCCACAGGGGCTTCCAGAGAC
C5276HH080-4.seq(1>3369)                                   →  AACACAGAAGCCTGCTTTCTGGTAGAGCTACCCAGTTTACAGGGGCCACAGGCGCTATCATGACCACTGAAACCACGAAGACCGCAAGGGCCACAGGGGCTTCCAGAGAC
E05-PD64524-C5276HH080-4-2-SEQ3FNP.ab1(1>751)              →  AACACAGAAGCCTGCTTTCTGGTAGAGCTACCCAGTTTACAGGGGCCACAGGCGCTATCATGACCACTGAAACCACGAAGACCGCAAGGGCCACAGGGGCTTCCAGAGAC

                                                              2320      2330      2340      2350      2360      2370      2380      2390      2400      2410      2420
                                                              ATGGCCGGAGCTCAGGCAGCTGCTGTTGCACTGAACGAAGAATTCTTAAGAAATTATTTCACTGATAAAGCGGCCTCTTACACTGAGGAAGATGAAAATCACACAGCCAA
C5276HH080-4.seq(1>3369)                                   →  ATGGCCGGAGCTCAGGCAGCTGCTGTTGCACTGAACGAAGAATTCTTAAGAAATTATTTCACTGATAAAGCGGCCTCTTACACTGAGGAAGATGAAAATCACACAGCCAA
E05-PD64524-C5276HH080-4-2-SEQ3FNP.ab1(1>751)              →  ATGGCCGGAGCTCAGGCAGCTGCTGTTGCACTGAACGAAGAATTCTTAAGAAATTATTTCACTGATAAAGCGGCCTCTTACACTGAGGAAGATGAAAATCACACAGCCAA

                                                              2430      2440      2450      2460      2470      2480      2490      2500      2510      2520      2530
                                                              AGATTGCCTTCTGGTTTATTCTCAGGAAGAAACTGAATCGCTGAATGCTTCTATTGGTTGTTGCAGTTTTATTGAAGGAGAGCTAGATGACCGCTTCTTAGATGATTTGG
C5276HH080-4.seq(1>3369)                                   →  AGATTGCCTTCTGGTTTATTCTCAGGAAGAAACTGAATCGCTGAATGCTTCTATTGGTTGTTGCAGTTTTATTGAAGGAGAGCTAGATGACCGCTTCTTAGATGATTTGG
E05-PD64524-C5276HH080-4-2-SEQ3FNP.ab1(1>751)              →  AGATTGCCTTCTGGTTTATTCTCAGGAAGAAACTGAATCGCTGAATGCTTCTATTGGTTGTTGCAGTTTTATTGAAGGAGAGCTAGATGACCGCTTCTTAGATGATTTGG

                                                              2540      2550      2560      2570      2580      2590      2600      2610      2620      2630      2640
                                                              GACTTAAATTCAAGACACTAGCTGAAGTTTGCCTGGGTCAAAAAATAGATATAAATAAGGAAATTGAGCAGAGACAAAAACCTGCCACAGAAACAAGTATGAACACAGCT
C5276HH080-4.seq(1>3369)                                   →  GACTTAAATTCAAGACACTAGCTGAAGTTTGCCTGGGTCAAAAAATAGATATAAATAAGGAAATTGAGCAGAGACAAAAACCTGCCACAGAAACAAGTATGAACACAGCT
E05-PD64524-C5276HH080-4-2-SEQ3FNP.ab1(1>751)              →  GACTTAAATTCAAGACACTAGCTGAAGTTTGCCTGGGTCAAAAAATAGATATAAATAAGGAAATTGAGCAGAGACAAAAACCTGCCACAGAAACAAGTATGAACACAGCT
```

```
                                              2650      2660      2670      2680      2690      2700      2710      2720      2730      2740      2750
                                              TCACATTCACTCTGTGAGCAAACTATGGTTAATTCAGAGAATACCTACTCCTCTGGCAGTAGCTTCCCAGTTCCAAAATCTTTGCAAGAAGCCAATGCAGAGAAAGTAAC
C5276HH080-4.seq(1>3369)                    → TCACATTCACTCTGTGAGCAAACTATGGTTAATTCAGAGAATACCTACTCCTCTGGCAGTAGCTTCCCAGTTCCAAAATCTTTGCAAGAAGCCAATGCAGAGAAAGTAAC
E05-PD64524-C5276HH080-4-2-SEQ3FNP.ab1(1>751) → TCACATTCACTCTGTGAGCAAACTATGGTTAATTCAGAGAATACCTACTCCTCTGGCAGTAGCTTCCCAGTTCCAAAATCTTTGCAAGAAGCCAATGCAGAGAAAGTAAC
E06-PD64524-C5276HH080-4-2-SEQ4FNP.ab1(1>764) → TAGCTTCCCAGTTCCAAAATCTTTGCAAGAAGCCAATGCAGAGAAAGTAAC

                                              2760      2770      2780      2790      2800      2810      2820      2830      2840      2850      2860
                                              TCAGGAAATAGTCACTGAAAGATCTGTGTCTTCTAGGCAGGCGCAAAAGGTAGCTACACCTCTTCCTGACCCAATGGCTTCTAGAAATGTGATAGCAACAGAAACTTCCT
C5276HH080-4.seq(1>3369)                    → TCAGGAAATAGTCACTGAAAGATCTGTGTCTTCTAGGCAGGCGCAAAAGGTAGCTACACCTCTTCCTGACCCAATGGCTTCTAGAAATGTGATAGCAACAGAAACTTCCT
E05-PD64524-C5276HH080-4-2-SEQ3FNP.ab1(1>751) → TCAGGAAATAGTCACTGAAAGATCTGTGTCTTCTAGGCAGGCGCAAAAGGTAGCTACACCTCTTCCTG
E06-PD64524-C5276HH080-4-2-SEQ4FNP.ab1(1>764) → TCAGGAAATAGTCACTGAAAGATCTGTGTCTTCTAGGCAGGCGCAAAAGGTAGCTACACCTCTTCCTGACCCAATGGCTTCTAGAAATGTGATAGCAACAGAAACTTCCT
E07-PD64524-C5276HH080-4-2-SEQ5FNP.ab1(1>767) → AATGGCTTCTAGAAATGTGATAGCAACAGAAACTTCCT

                                              2870      2880      2890      2900      2910      2920      2930      2940      2950      2960      2970
                                              ATGTCACAGGGTCCACTATGCCACCAACCACTGTGATCCTGGGTCCTAGCCAGCCACAGAGCCTTATTGTGACAGAGAGGGTGTATGCTCCAGCTTCTACCTTGGTAGAT
C5276HH080-4.seq(1>3369)                    → ATGTCACAGGGTCCACTATGCCACCAACCACTGTGATCCTGGGTCCTAGCCAGCCACAGAGCCTTATTGTGACAGAGAGGGTGTATGCTCCAGCTTCTACCTTGGTAGAT
E06-PD64524-C5276HH080-4-2-SEQ4FNP.ab1(1>764) → ATGTCACAGGGTCCACTATGCCACCAACCACTGTGATCCTGGGTCCTAGCCAGCCACAGAGCCTTATTGTGACAGAGAGGGTGTATGCTCCAGCTTCTACCTTGGTAGAT
E07-PD64524-C5276HH080-4-2-SEQ5FNP.ab1(1>767) → ATGTCACAGGGTCCACTATGCCACCAACCACTGTGATCCTGGGTCCTAGCCAGCCACAGAGCCTTATTGTGACAGAGAGGGTGTATGCTCCAGCTTCTACCTTGGTAGAT

                                              2980      2990      3000      3010      3020      3030      3040      3050      3060      3070      3080
                                              CAGCCTTATGCTAATGAAGGTACAGTTGTGGTCACTGAAAGAGTAATACAGCCTCATGGGGGTGGATCGAATCCTCTGGAAGGCACTCAGCATCTTCAAGATGTACCTTA
C5276HH080-4.seq(1>3369)                    → CAGCCTTATGCTAATGAAGGTACAGTTGTGGTCACTGAAAGAGTAATACAGCCTCATGGGGGTGGATCGAATCCTCTGGAAGGCACTCAGCATCTTCAAGATGTACCTTA
E06-PD64524-C5276HH080-4-2-SEQ4FNP.ab1(1>764) → CAGCCTTATGCTAATGAAGGTACAGTTGTGGTCACTGAAAGAGTAATACAGCCTCATGGGGGTGGATCGAATCCTCTGGAAGGCACTCAGCATCTTCAAGATGTACCTTA
E07-PD64524-C5276HH080-4-2-SEQ5FNP.ab1(1>767) → CAGCCTTATGCTAATGAAGGTACAGTTGTGGTCACTGAAAGAGTAATACAGCCTCATGGGGGTGGATCGAATCCTCTGGAAGGCACTCAGCATCTTCAAGATGTACCTTA

                                              3090      3100      3110      3120      3130      3140      3150      3160      3170      3180      3190
                                              CGTCATGGTGAGGGAAAGAGAGAGCTTCCTTGCCCCCAGCTCAGGTGTGCAGCCTACTCTGGCCATGCCTAATATAGCAGTAGGACAGAATGTGACAGTGACAGAAAGAG
C5276HH080-4.seq(1>3369)                    → CGTCATGGTGAGGGAAAGAGAGAGCTTCCTTGCCCCCAGCTCAGGTGTGCAGCCTACTCTGGCCATGCCTAATATAGCAGTAGGACAGAATGTGACAGTGACAGAAAGAG
E06-PD64524-C5276HH080-4-2-SEQ4FNP.ab1(1>764) → CGTCATGGTGAGGGAAAGAGAGAGCTTCCTTGCCCCCAGCTCAGGTGTGCAGCCTACTCTGGCCATGCCTAATATAGCAGTAGGACAGAATGTGACAGTGACAGAAAGAG
E07-PD64524-C5276HH080-4-2-SEQ5FNP.ab1(1>767) → CGTCATGGTGAGGGAAAGAGAGAGCTTCCTTGCCCCCAGCTCAGGTGTGCAGCCTACTCTGGCCATGCCTAATATAGCAGTAGGACAGAATGTGACAGTGACAGAAAGAG

                                              3200      3210      3220      3230      3240      3250      3260      3270      3280      3290      3300
                                              TTCTAGCACCTGCTTCCACTCTGCAATCCAGTTACCAGATTCCCACTGAAAATTCTATGACGGCTAGGAACACCACGGTGTCTGGAGCTGGAGTCCCTGGCCCTCTGCCA
C5276HH080-4.seq(1>3369)                    → TTCTAGCACCTGCTTCCACTCTGCAATCCAGTTACCAGATTCCCACTGAAAATTCTATGACGGCTAGGAACACCACGGTGTCTGGAGCTGGAGTCCCTGGCCCTCTGCCA
E06-PD64524-C5276HH080-4-2-SEQ4FNP.ab1(1>764) → TTCTAGCACCTGCTTCCACTCTGCAATCCAGTTACCAGATTCCCACTGAAAATTCTATGACGGCTAGGAACACCACGGTGTCTGGAGCTGGAGTCCCTGGCCCTCTGCCA
E07-PD64524-C5276HH080-4-2-SEQ5FNP.ab1(1>767) → TTCTAGCACCTGCTTCCACTCTGCAATCCAGTTACCAGATTCCCACTGAAAATTCTATGACGGCTAGGAACACCACGGTGTCTGGAGCTGGAGTCCCTGGCCCTCTGCCA

                                              3310      3320      3330      3340      3350      3360      3370      3380      3390      3400      3410
                                              GATTTTGGTTTAGAGGAATCTGGTCATTCTAATTCTACCATAACCACATCTTCCACCAGAGTTACCAAGCATAGCACTGTACAGCATTCTTACTCCTAAGGATCCACCGG
C5276HH080-4.seq(1>3369)                    → GATTTTGGTTTAGAGGAATCTGGTCATTCTAATTCTACCATAACCACATCTTCCACCAGAGTTACCAAGCATAGCACTGTACAGCATTCTTACTCCTAAGGATCC
E06-PD64524-C5276HH080-4-2-SEQ4FNP.ab1(1>764) → GATTTTGGTTTAGAGGAATCTGGTCATTCTAATTCTACCATAACCACATCTTCCACCAGAGTTACCAAGCATAGCACTGTACAGCATTCTTACTCCTAAGGATCCACCGG
E07-PD64524-C5276HH080-4-2-SEQ5FNP.ab1(1>767) → GATTTTGGTTTAGAGGAATCTGGTCATTCTAATTCTACCATAACCACATCTTCCACCAGAGTTACCAAGCATAGCACTGTACAGCATTCTTACTCCTAAGGATCCACCGG
```

```
                                                         3420      3430      3440      3450      3460      3470      3480      3490      3500      3510      3520
                                                ATCTAGATAACTGATCATAATCAGCCATACCACATTTGTAGAGGTTTTACTTGCTTTAAAAAACCTCCCACACCTCCCCCTGAACCTGAAACATAAAATGAATGCAATTG
E06-PD64524-C5276HH080-4-2-SEQ4FNP.ab1(1>764)  → ATCTAGATAACTGATCATAATCAGCCATACCACATTTGTAGAGGTTTTACTTG
E07-PD64524-C5276HH080-4-2-SEQ5FNP.ab1(1>767)  → ATCTAGATAACTGATCATAATCAGCCATACCACATTTGTAGAGGTTTTACTTGCTTTAAAAAACCTCCCACACCTCCCCCTGAACCTGAAACATAAAATGAATGCAATTG

                                                         3530      3540      3550      3560      3570      3580
                                                TTGTTGTTAACTTGTTTATTGCAGCTTATAATGGTTACAAATAAAGCAATAGCATCACAAATTTCACAA
E07-PD64524-C5276HH080-4-2-SEQ5FNP.ab1(1>767)  → TTGTTGTTAACTTGTTTATTGCAGCTTATAATGGTTACAAATAAAGCAATAGCATCACAAATTTCACAA
```
